# Correction: Unimproved source of drinking water and the associated factors: Insights from the 2020 Somalia demographic and health survey

**DOI:** 10.1371/journal.pgph.0006215

**Published:** 2026-03-30

**Authors:** Abdisalam Mahdi Hassan, Nimo Mohamoud Barakale, Omran Salih, Abdisalam Hassan Muse

The second affiliation for the second author is not indicated. Omran Salih is also affiliated with #1: School of Postgraduate Studies and Research, Amoud University, Borama, Somaliland.
