# Supplementary material for: Unimproved source of drinking water and the associated factors: Insights from the 2020 Somalia demographic and health survey
Source: PLOS Glob Public Health. 2024 Dec 5;4(12):e0003844. doi: 10.1371/journal.pgph.0003844 (PMC11620610; doi:10.1371/journal.pgph.0003844)
Supplement: Attachment — Submitted filename: Response To Reviewers.docx [file pgph.0003844.s001.docx]

**PLOS Global Public Health**

**Manuscript ID: PGPH-D-24-01613**

**Manuscript Title: Unimproved Source of Drinking Water and its Associated Factors: Insights from Somalia Health and Demographic Survey 2020.**

**Dear Respected Professor Editor**

Thank you for your email dated Wed, Sep 4, 2024 at 4:15 AM, and for the reviewer’s comments.

We start by thanking the reviewers for their careful reading of our paper and for helping us to improve its quality and presentation.

We have carefully considered each of their comments and have made comprehensive revisions to improve the clarity and quality of our work.

The updated manuscript and the point-by-point response document with tracked changes are attached for your review.

**REVIEWER #1**

The manuscript has no issues about dual publication, research ethics, or publication ethics.

We appreciate your time and effort in reviewing our work. We have carefully considered your comments and have made all the proposed revisions

However, there are some minor issues to be addressed. The author should address the following issues:
1. The definition or criteria for improved and unimproved sanitation facilities

We have added a clear definition of both improved and unimproved water sources as our outcome variable is looking the source of drinking water not sanitation facilities (See Page 1)

2. The author indicated that variables with p-values less than 0.4 in the multivariate model were fitted in the bivariate analysis. Why was less than 0.4 was used?

The decision to include variables with p-values less than 0.4 in the multivariate analysis due to

- **Minimizing Type II Error:** A more liberal threshold in the bivariate analysis helps to avoid excluding potentially important variables that might have a significant association in the multivariable model due to collinearity or other complexities.
- **Controlling for Multicollinearity:** By including variables with p-value < 0.4 in the multivariate model, we can explore the individual and combined effects of variables while controlling for potential collinearity.

However, since no variables in our bivariate analysis had p-values greater than 0.4, we did not need to exclude any from the multivariate analysis. Therefore, the initial assumption regarding variable exclusion was deemed unnecessary and has been removed from the Data Management and Statistical Analysis section (See Page 4).

3. The author should address a few grammatical error issues. For instance, was used instead of were, Can have got more, etc.)

We have carefully reviewed the manuscript for grammatical errors and have corrected the instances you identified.
4.Six (6) references are not enough for such research.

You are correct that our initial reference list was insufficient. We have expanded the reference list to include relevant literature on water access, sanitation, and public health in Somalia and other African countries. We have added more references, bringing the total to twenty-one. We believe this expanded reference list provides a stronger foundation for our research and supports our arguments.

**REVIEWER #2**

The study has a commendable objective of attempting to explore factors associated with unimproved drinking water sources in Somalia. However, some of the major concerns with the submitted manuscript include:

1. It is not well formatted. The manuscript has sections that needs to be rewritten for clarity.

We appreciate the reviewer's feedback regarding the formatting of the manuscript. We have revised the manuscript for clarity and readability, restructuring sections, clarifying language, and adding headings and subheadings as suggested

2. The discussion section is not thoroughly informed by the presented exploratory analysis.

We have thoroughly revised the discussion section to directly link key findings from the tables to existing literature and highlight significant relationships between variables (See Page 10).

3. The study does not sufficiently engage existing literature or previous works (or may have not accurately cited all of the sources used) hence only 6 articles are referenced in the reference section.

We acknowledge the reviewer's point about the limited literature review. We have expanded the literature review, including more relevant studies related to drinking water access in Somalia and similar contexts, and carefully reviewed our references to ensure accuracy (See Page 1 and 2)

4. The study does not sufficiently address practical implications or recommendations for its discussed findings.

We have incorporated a dedicated section on policy implications, presenting specific recommendations for policymakers and stakeholders based on our findings. This includes outlining actionable steps to address the identified challenges (See Page 11).

**REVIEWER #3**

Original and pertinent work using robust methodology.

1. The recommendation in concluding section is generic and does not clearly link to study findings.

We have revised the concluding section to directly connect the recommendations to specific findings of the study. This includes providing more detailed and tailored suggestions based on the data we analyzed (See page 11).

2. Both authors state contribution to data the collection, but it’s not clear if they participated in primary study.

We would like to clarify that the previous statement mentioning our involvement in data collection was an oversight. We did not participate in the primary data collection for this study. Instead, we have conducted a rigorous analysis of publicly available data from the Somalia DHS to generate new insights and findings (See page 12).

3. Authors should verify, ethical approval process of the primary study and affirm that standard ethical principles were followed rather than an assumption that it “likely” followed the same.

We have verified the ethical approval process of the Somalia DHS study and confirmed that it adheres to standard ethical principles. We have included this information in the manuscript (Page 4).

**Reviewer #4**

 Manuscript is well coordinated however there are a few points to consider.

1. Manuscript is well coordinated however there are a few points to consider. Please refer to review submitted. Please re look at manuscript for grammatical errors and areas where lowercase and uppercase letters has been used.

Unfortunately, the reviewer comments have not been found. However, We have meticulously reviewed the manuscript for grammatical errors and inconsistencies in capitalization. We have ensured that the text is clear, concise, and adheres to proper grammar and formatting conventions.

**Reviewer #5**

The manuscript presents an important topic in the field of global public health. This document outlines a study examining the factors associated with the use of unimproved drinking water sources in Somalia, based on data from the 2020 Somalia Health and Demographic Survey (SHDS). The study highlights that approximately 22.04% of the Somali population relies on unimproved water sources, with significant disparities based on household characteristics, such as age, education level, residence, region, wealth index, and employment status.
The manuscript is generally well-written, but there are areas where clarity could be improved.

1. Some sections, particularly the methods and discussion, are dense and could benefit from more concise language.

We have revised the Methods and Discussion sections to ensure they are more concise and easy to understand. We have removed redundancies and streamlined the language to enhance clarity for the reader.

2. Additionally, some figures and tables are difficult to interpret and would be more effective with clearer labels and explanations.

We have revised the figures and tables to improve their clarity and interpretability. We have added clearer labels, explanations, and annotations where necessary.

3. The manuscript briefly mentions ethical approval, but there is no discussion of how informed consent was obtained from participants. Given the sensitive nature of the study topic, it is crucial to provide more information on the ethical procedures followed to ensure participant safety and confidentiality.

We have expanded our discussion on ethical considerations to include information on how informed consent was obtained from participants in the 2020 SHDS, which is the primary data source for our study. The SHDS adheres to strict ethical guidelines, including obtaining informed consent from participants. This information is now clearly presented in the manuscript.

4. I recommend the authors address the methodological issues raised, particularly regarding the sample size and data analysis.

We have addressed the methodological issues raised regarding sample size and data analysis, providing further clarification and justification for the methodology employed.

5. A more cautious interpretation of the results would also strengthen the manuscript.

We have adopted a more cautious approach to interpreting the results, highlighting the limitations of the study and acknowledging potential areas for further research.

6. Clarifying the ethical considerations and improving the presentation of data will help ensure the study's impact on the field.

We believe the improvements made in ethical considerations and data presentation will strengthen the study's impact on the field, ensuring it is ethically sound and clearly communicates its findings.

**Reviewer #6**

1. Introduction: The introduction is somewhat cluttered with information and could benefit from clearer organization. The background information on global water access and the SDG goals is useful, but the connection to Somalia’s specific context could be strengthened. The narrative would be clearer if it flowed from the global context to the specific challenges in Somalia, leading into the study’s objectives.

We have reorganized the introduction to provide a more focused narrative that connects global water access issues to the specific challenges in Somalia. This leads naturally into the study's objectives and strengthens the connection to the Somali context.

2. Results Section: The presentation of results is dense and could be streamlined for better readability. The inclusion of too many details in the text, such as the exact percentages and odds ratios is overwhelming. Consider summarizing key findings in a more digestible format, perhaps using bullet points or summary tables for major results, and focusing the narrative on interpreting these findings.

We have streamlined the presentation of results to improve readability. We have condensed the text by summarizing key findings in bullet points and summary tables, focusing the narrative on interpreting the findings rather than overwhelming the reader with granular details.
3. Interpretation of Findings: The discussion does not fully explore the implications of the findings. For example, the higher likelihood of unimproved water sources in female-headed households is noted, but the underlying reasons for this disparity are not sufficiently explored. Additionally, the manuscript could benefit from a more in-depth discussion on why certain regions, such as Mudug and Nugaal, show different patterns compared to others. The conclusion mentions that Somalia should enhance its provision of improved water sources but does not provide specific recommendations or strategies on how this could be achieved, given the identified factors.

We have expanded the discussion to explore the implications of the findings more deeply. We have addressed the reasons behind the higher likelihood of unimproved water sources in female-headed households, delving into potential underlying factors. We have also provided a more in-depth discussion on the variations observed in different regions, analyzing potential explanations for these differences.
4. Inconsistencies in Reporting: There are some inconsistencies in the reporting of results, particularly in the regression analysis. For instance, the manuscript states that "the odds of unemployed household heads are 1.14 times more likely to use unimproved sources of drinking water," but it is unclear whether this association is statistically significant. Clearer reporting of significance levels and confidence intervals throughout the results section would improve clarity.

We have ensured consistency in reporting results, particularly in the regression analysis. We have clearly reported significance levels (p-values) and confidence intervals for all statistically significant findings, enhancing the clarity of the results section.

4. Lack of Policy Implications: While the study identifies several factors associated with the use of unimproved water sources, the practical implications of these findings for policy and intervention strategies are not well developed. The paper would be stronger if it included more detailed recommendations for policymakers on how to address these issues, particularly in the Somali context.
We have dedicated a robust section to discussing the practical implications of the findings for policy and intervention strategies in the Somali context. We have included detailed recommendations for policymakers on how to address the identified issues, providing specific strategies for improving water access.

Recommendations:
1. Reorganize the Introduction: Streamline the introduction to provide a clearer narrative that connects global water access issues to the specific challenges in Somalia, leading naturally into the study's objectives.
2. Enhance the Discussion: Expand on the discussion of findings, particularly regarding the socio-cultural and economic factors that may explain the disparities in water access. Provide more detailed policy recommendations based on the study’s findings.
3. Simplify the Results Presentation: Consider summarizing the key findings in a more accessible format and focusing the narrative on the interpretation rather than the granular details of the data.
4. Improve Reporting Consistency: Ensure that all significant results are clearly reported with appropriate p-values and confidence intervals. Address any inconsistencies in the statistical reporting.
5. Expand Policy Implications: Develop a more robust section on the practical implications of the findings, offering specific recommendations for how Somalia can improve water access, taking into account the identified factors.

We appreciate Reviewer #6's thoughtful comments and acknowledge the areas for improvement they have highlighted. We have carefully addressed each point raised and incorporated the suggested changes.
