# Supplementary material for: Unimproved source of drinking water and the associated factors: Insights from the 2020 Somalia demographic and health survey
Source: PLOS Glob Public Health. 2024 Dec 5;4(12):e0003844. doi: 10.1371/journal.pgph.0003844 (PMC11620610; doi:10.1371/journal.pgph.0003844)
Supplement: Attachment — Submitted filename: PGPH-D-24-01613_R1 Rereview.docx [file pgph.0003844.s002.docx]

PLOS Global Public Health

**Unimproved Source of Drinking Water and its Associated Factors: Insights from Somalia Health and Demographic Survey 2020.**

--Manuscript Draft--

| **Manuscript Number:** | PGPH-D-24-01613R1 |
| --- | --- |
| **Article Type:** | Research Article |
| **Full Title:** | Unimproved Source of Drinking Water and its Associated Factors: Insights from Somalia Health and Demographic Survey 2020. |
| **Short Title:** | Unimproved Drinking Water Sources Among Somali Households |
| **Corresponding Author:** | Omran Salih, Ph.D. Amoud University Borama, Awdal SOMALIA |
| **Order of Authors:** | Abdisalam Mahdi, MSc |
|  | Nimo Mohamoud Barakale |
|  | Omran Salih |
|  | Abdisalam Hassan Muse |
| **Keywords:** | Unimproved water sources, multivariate analysis, SHDS, Somalia. |
| **Abstract:** | Background: Access to safe drinking water is a fundamental human right and a critical public health concern, particularly in lower- and middle-income countries with limited infrastructure. Somalia faces significant challenges in providing improved drinking water sources, with a high prevalence of unimproved sources. This study analyzes data from the 2020 Somalia Health and Demographic Survey to investigate the prevalence of unimproved drinking water sources and identify associated factors.  Methods: A cross-sectional analysis of data from the 2020 Somalia Health and Demographic Survey (SHDS) was conducted, involving 32,300 participants.  Multivariable logistic regression, implemented in Stata 16 software, was used to identify factors associated with using an unimproved drinking water source. Variables with a p-value < 0.05 in bivariate analysis were included in the multivariate model.  Adjusted odds ratios (AOR) and 95% confidence intervals (CI) were used to estimate the association of significant variables with the outcome.  Results: Approximately 22.04% (95% CI: 21.5%, 22.4%) of the Somali population utilizes unimproved drinking water sources. Households with a head aged 20 years and above (AOR = 0.88, p = 0.059) were less likely to rely on unimproved sources compared to households with a head under 20 years. Female household heads (AOR  = 1.17, p = 0.000) were more likely to use unimproved sources than male household heads. Unemployed partners (AOR = 1.14, p = 0.000) were more likely to use unimproved sources than employed partners. Rural (AOR = 1.12, p = 0.013) and nomadic (AOR = 0.93, p = 0.175) residents were more likely to use unimproved sources compared to urban residents. Households in Mudug (AOR = 31.18, p = 0.000), Nugaal (AOR = 4.15, p = 0.000), Bari (AOR = 5.26, p = 0.000), and Sanaag (AOR =  2.52, p = 0.000) regions were less likely to use unimproved sources compared to households in other regions.  Conclusion: The prevalence of unimproved drinking water sources in Somalia is substantial, exceeding proportions reported in some other African countries. These findings highlight the urgent need for Somalia to improve its provision of safe and accessible water sources, particularly for vulnerable populations. |
| **Additional Information:** |  |
| **Question** | **Response** |
| **Financial Disclosure** | The authors received no specific funding for this work |

| Enter a financial disclosure statement that describes the sources of funding for the work included in this submission and the role the funder(s) played. This includes grants and any commercial funding of the work or authors.  This statement will be typeset if the manuscript is accepted for publication.  *Please review the* [*submission guidelines*](http://journals.plos.org/globalpublichealth/s/submission-guidelines#loc-financial-disclosure-statement) *and the instructions link below for detailed requirements and guidance.* |  |
| --- | --- |
| **Competing Interests**  On behalf of all authors, disclose any competing interests that could be perceived to bias this work.  This statement will be typeset if the manuscript is accepted for publication.  *Please review the instructions link below and PLOS Global Public Health’s* [*competing interests*](https://journals.plos.org/globalpublichealth/s/competing-interests) *policy to determine what information must be disclosed at submission.* | The authors have declared that no competing interests exist. |
| **Data Availability**  Before publication, Authors are required to make fully available and without restriction all data underlying their findings. Please see our [PLOS Data](https://journals.plos.org/globalpublichealth/s/data-availability) [Policy](https://journals.plos.org/globalpublichealth/s/data-availability) page for detailed information on this policy.  A **Data Availability Statement**, detailing where the data can be accessed, is required at first submission. Insert your Data Availability Statement in the box below. | This study used secondary data from the Somalia Health and Demographic Survey (SHDS) 2020, which is publicly available on the website (https://microdata.nbs.gov.so/index.php/catalog/50). Access was granted after registration using an institutional email [(abdisalam.hassan@amoud.edu.so).](mailto:(abdisalam.hassan@amoud.edu.so) As the data is publicly available and anonymized, formal ethical approval from an Institutional Review Board (IRB) or Ethics Committee was not required for our study. However, it is important to acknowledge the ethical considerations during the original SHDS 2020 data collection. The National Bureau of Statistics (NBS), responsible for conducting the SHDS, likely has an established IRB or Ethics Committee that reviewed and approved the survey design and data collection procedures. The statement mentions that enumerators received training on obtaining informed consent (likely verbal) from participants, ensuring confidentiality, and building rapport. This aligns with ethical research practices for human subjects. Further details on the original SHDS 2020 IRB approval process or specific consent procedures could be obtained by contacting the NBS. |

| Please see the [data reporting](https://journals.plos.org/globalpublichealth/s/submission-guidelines#loc-data-reporting) section of our submission guidelines for instructions on what you need to include in your Data Availability Statement.  This statement will be typeset if the manuscript is accepted for publication.  *PLOS allows rare exemptions to address legal and ethical concerns. If you have legal or ethical restrictions, please use the box below to detail these in full sentences for the Journal team to consider.* |  |
| --- | --- |

<https://www2.cloud.editorialmanager.com/pgph/download.aspx?id=431260&guid=51553b51-7d53-49b8-a532-bdbc4df2491a&scheme=1>Manuscript	[Click here to](https://www2.cloud.editorialmanager.com/pgph/download.aspx?id=431260&guid=51553b51-7d53-49b8-a532-bdbc4df2491a&scheme=1)

[access/download;Manuscript;Manuscript_OS_V1.docx](https://www2.cloud.editorialmanager.com/pgph/download.aspx?id=431260&guid=51553b51-7d53-49b8-a532-bdbc4df2491a&scheme=1)

# 1 Unimproved Source of Drinking Water and its Associated Factors: Insights from Somalia

2 **Health and Demographic Survey 2020.**
3 Abdisalam Mahdi Hassan, Nimo Mohamoud Barakale, Omran Salih, Abdisalam Hassan Muse
4 School of Postgraduate Studies and Research, Amoud University, Borama 25263, Somalia
5 **Corresponding email:** [omran.salih@amoud.edu.so](mailto:omran.salih@amoud.edu.so)

# 6 Abstract

7 **Background:** Access to safe drinking water is a fundamental human right and a critical public health concern,
8 particularly in lower- and middle-income countries with limited infrastructure. Somalia faces significant challenges
9 in providing improved drinking water sources, with a high prevalence of unimproved sources. This study analyzes
10 data from the 2020 Somalia Health and Demographic Survey to investigate the prevalence of unimproved drinking
11 water sources and identify associated factors.
12 **Methods:** A cross-sectional analysis of data from the 2020 Somalia Health and Demographic Survey (SHDS) was
13 conducted, involving 32,300 participants. Multivariable logistic regression, implemented in Stata 16 software, was
14 used to identify factors associated with using an unimproved drinking water source. Variables with a p-value < 0.05
15 in bivariate analysis were included in the multivariate model. Adjusted odds ratios (AOR) and 95% confidence
16 intervals (CI) were used to estimate the association of significant variables with the outcome.
17 **Results:** Approximately 22.04% (95% CI: 21.5%, 22.4%) of the Somali population utilizes unimproved drinking
18 water sources. Households with a head aged 20 years and above (AOR = 0.88, p = 0.059) were less likely to rely on
19 unimproved sources compared to households with a head under 20 years. Female household heads (AOR = 1.17, p =
20 0.000) were more likely to use unimproved sources than male household heads. Unemployed partners (AOR = 1.14,
21 p = 0.000) were more likely to use unimproved sources than employed partners. Rural (AOR = 1.12, p = 0.013) and
22 nomadic (AOR = 0.93, p = 0.175) residents were more likely to use unimproved sources compared to urban residents.
23 Households in Mudug (AOR = 31.18, p = 0.000), Nugaal (AOR = 4.15, p = 0.000), Bari (AOR = 5.26, p = 0.000),
24 and Sanaag (AOR = 2.52, p = 0.000) regions were less likely to use unimproved sources compared to households in
25 other regions.
26 **Conclusion:** The prevalence of unimproved drinking water sources in Somalia is substantial, exceeding proportions
27 reported in some other African countries. These findings highlight the urgent need for Somalia to improve its provision
28 of safe and accessible water sources, particularly for vulnerable populations.
29 **Keywords:** Unimproved water sources, multivariate analysis, SHDS, Somalia.

## 30

31

#### 32 Introduction

33 Access to clean water is a fundamental human right, essential for human development, growth, and well-
34 being (Andualem et al., 2021). It is a crucial element in achieving sustainable development, as highlighted by
35 Sustainable Development Goal (SDG) 6, which explicitly addresses water and sanitation with the target of providing
36 universal and equitable access to safe and affordable drinking water for all by 2030 (Alfven et al., 2014). The long-
37 term objective is to ensure that everyone has access to safe water at home, ideally within a 30-minute round trip
38 (Belay & Andualem, 2022; Desye et al., 2023). However, globally, over 2 billion people still lack access to safely
39 managed drinking water services (Win et al., 2023).
40 The burden of inadequate water access disproportionately affects low- and middle-income countries,
41 particularly in sub-Saharan Africa, where infrastructure limitations, poverty, and conflict often impede the provision
42 of safe water sources (Belay & Andualem, 2022). The majority of households with unimproved water sources are found
43 in sub-Saharan Africa, often requiring long journeys to collect water (Belay & Andualem, 2022). This lack of safe
44 water access contributes to increased risks of infectious diseases, including cholera, typhoid, schistosomiasis, and
45 infections of the respiratory, skin, and eye systems (Andualem et al., 2021).
46 Somalia, a country located in sub-Saharan Africa, faces significant challenges in providing safe drinking
47 water to its population (Farih et al., 2024; Hassan et al., 2024; SNBS, 2020). Despite the national target of achieving
48 universal access to an improved source of drinking water outlined in the Somalia National Development Plan (NDP),
49 the population in Somalia has not yet been able to reach this goal (Tsegaw et al., 2023; Water et al., 2018; Win et al.,
50 2023). In Somalia, slightly over three-quarters (76%) of urban households have access to improved water sources,
51 compared to just over half (55%) of rural households and 35% of nomadic households (DNS Government of Somalia,
52 2020). This disparity in water access has serious implications for the health and well-being of Somali communities,
53 leading to increased vulnerability to waterborne diseases and hindering progress towards achieving national
54 development goals.
55 The lack of comprehensive research on the prevalence and associated factors of unimproved drinking water
56 sources in Somalia represents a significant research gap (Belay & Andualem, 2022). Existing data suggests that 35%
57 of Somali households rely on unimproved sources for drinking water (DNS Government of Somalia, 2020),
58 highlighting the need for a deeper understanding of this issue. Previous studies have examined factors influencing
59 water access in other African countries, providing valuable insights into the complex factors at (Amoak et al., 2023;
60 Essuman et al., 2023; Omidakhsh & von Ehrenstein, 2021; Oppong et al., 2022; Sahiledengle et al., 2021). However,
61 a comprehensive investigation using nationally representative data on unimproved sources in Somali households is
62 lacking.
63 This study aims to fill this critical research gap by analyzing data from the 2020 Somalia Health and
64 Demographic Survey (SHDS) to investigate the prevalence of unimproved drinking water sources and identify
65 associated factors. The study's findings could assist policymakers in implementing measures to reduce the
66 consequences of utilizing water from unimproved sources in Somalia and work towards achieving the national goal
67 of universal access to safe water.
68 **Material and Methods**

#### 69 Study Design

70 This cross-sectional study utilized secondary data from the 2020 Somalia Health and Demographic Survey
71 (SHDS), the first nationally representative household survey conducted in Somalia. The SHDS is a crucial resource
72 for strengthening national data systems and promoting evidence-based planning for improved public health outcomes.

#### 73 Study Setting

74 The study focused on Somalia, a country located in the Horn of Africa with an estimated population of 12.3
75 million. Somalia boasts the longest coastline in Africa, stretching 3,333 kilometers along the Indian Ocean and the
76 Gulf of Aden. It shares borders with Ethiopia to the west, Kenya to the southwest, and Djibouti to the northwest.

#### 77 Data source

78 This study utilized the 2020 SHDS dataset, collected between 2018 and 2020. The data was obtained from
79 the DHS website: [www.dhsprogram.com.](http://www.dhsprogram.com/) Variables relevant to the study were extracted from the household record
80 (HR file) dataset. The SHDS sample was specifically designed to represent key indicators across various strata within
81 Somalia, achieving a response rate of 99%. This high response rate, encompassing urban, rural, and nomadic areas,
82 reflects the willingness and cooperation of participants. The final sample size for this study, after data cleaning and
83 the removal of missing variables, was 32,300 participants (Ali et al., 2024; Farih et al., 2024; Hassan et al., 2024).

#### 84 Study variables

85 **Dependent Variable**
86 The outcome variable for this study is the utilization of unimproved sources of drinking water. The drinking
87 water source was classified as unimproved if a household gets drinking water from an unprotected dug well,
88 unprotected spring, surface water, and tanker truck/cart with a small tank. For more clarification,
89 to define improved and unimproved drinking water sources, we utilize the classification system employed
90 in the DHS dataset. A household's primary drinking water source is categorized as improved if it falls within the
91 following categories: piped into dwelling or yard, public tap/standpipe, piped to neighbor, tube well/borehole,
92 protected well, protected spring, rainwater collection, tanker truck/cart, or bottled water. These sources generally
93 indicate a greater level of infrastructure, protection from contamination, and hygiene. Conversely, unimproved
94 sources, categorized as unprotected well, unprotected spring, or "other," are more susceptible to contamination due to
95 the lack of protective measures. It is important to note that even within these categories, the actual quality of the water
96 can vary depending on the specific context, level of maintenance, and overall hygiene practices. For the purposes of
97 our analysis, we utilize this classification system from the DHS dataset, acknowledging its inherent limitations and
98 the potential for variation in water quality within these broad categories.

## 99

100	**Independent Variables**

## 101

102 Individual and community-level independent variables were considered in this study. The age of the
103 household head (less than 20 years or 20 years and more), education status of the husband, whether he attended school
104 or not (yes or no), level of education of the respondent (no education, primary, secondary and higher), gender of the
105 household head (male or female), household head employment status (yes or no) and husband/partner work last year
106 (yes or no) were some of the individual-level factors included in this study. Community-level factors, residence (urban,
107 rural or nomadic), region (Awdal, Waqooyi galbeed, Togdheer, Sool, Sanaag, Bari, Nugaal, Mudug, Galgaduud,
108 Hiraan, Middle shabelle, Banadir, Bay, Bakool, Gedo and Lower juba) wealth index of the family (lowest, second,
109 middle, fourth and highest), family size members (less than four members and four or more members) and type of
110 sanitation facility (improved or unimproved) were considered.

## 111

112	**Data Management and Statistical Analysis**

## 113

114 Data analysis was conducted using Stata version 16. Following data cleaning and coding, descriptive analysis
115 was performed to determine the frequency and percentages of variables of interest. Due to the binary nature of the
116 outcome variable (improved vs. unimproved water sources), bivariate analysis utilizing chi-square testing was
117 employed to explore associations between the outcome variable and independent variables. Subsequently, multivariate
118 binary logistic regression was conducted to assess the relationship between the outcome variable and a set of
119 independent variables. Statistical significance was determined using an adjusted odds ratio (AOR) with a 95%
120 confidence interval (CI) and a p-value threshold of 0.05.

#### 121 Ethical Consideration

122 This study utilizes publicly available data from the Somalia Demographic and Health Survey (DHS)
123 program. The data, obtained from the official Somalia DHS website, has been anonymized and contains no personal
124 identifiers. As such, ethical approval was not required for this research. We adhere to the ethical principles of
125 respecting data privacy and ensuring the anonymity of individuals.

## 126

#### 127 Results

128 **Exploratory Analysis**
129 The study's findings, based on data from the 2020 Somalia Health and Demographic Survey (SHDS), revealed several
130 key factors associated with the use of unimproved drinking water sources. Univariate analysis (*Table 1*) showed that
131 a significant proportion of household heads (80.64%) were aged 20 years and older. Additionally, a large percentage
132 of respondents (87.02%) lacked formal education. The majority of household heads were male (66.40%), and a small
133 percentage were employed (1.14%). Respondents resided in urban (41.35%), rural (27.29%), and nomadic (31.36%)
134 areas. Notably, wealth status varied significantly among households, with the lowest (24.04%) and highest (16.46%)
135 wealth indices representing the extremes. Most households (77.30%) consisted of four or more members, and a
136 substantial proportion (59.70%) utilized unimproved sanitation facilities.
137 Table 1 Univariate analysis of both individual and community level factors associated with source of drinking water
138 using SHDS 2020. (n=32300)

| **Variable** | **Categories** | **Frequency (n)** | **Percentage %** |
| --- | --- | --- | --- |
| Age of household head | Less than 20 years | 6253 | 19.36 |
|  | 20 years and more | 26047 | 80.64 |
| Husband ever attended  school | Yes | 26985 | 83.54 |
|  | No | 5315 | 16.46 |
| Level of education of the respondent | No education | 28108 | 87.02 |
|  | Primary education | 3229 | 10 |
|  | Secondary education | 796 | 2.46 |
|  | Higher education | 167 | 0.52 |
| Sex of household head | Male | 21447 | 66.40 |
|  | Female | 10853 | 33.60 |
| House hold head  employment status | Yes | 367 | 1.14 |
|  | No | 31933 | 98.86 |
| Partner work | Yes | 13985 | 43.30 |
|  | No | 18315 | 56.70 |
| Residence | Urban | 13357 | 41.35 |
|  | Rural | 8814 | 27.29 |
|  | Nomadic | 10129 | 31.36 |
| Region | Awdal | 1333 | 4.13 |
|  | Waqoyigalbed | 2299 | 7.12 |
|  | Togdheer | 2798 | 8.66 |
|  | Sool | 2733 | 8.46 |
|  | Sanaag | 2727 | 8.44 |
|  | Bari | 1274 | 3.94 |
|  | Nugaal | 1498 | 4.64 |
|  | Mudug | 1490 | 4.61 |
|  | Galgadud | 1852 | 5.73 |
|  | Hiran | 2131 | 6.60 |
|  | Middle shabele | 1564 | 4.84 |
|  | Banadir | 4154 | 12.86 |

|  | Bay | 789 | 2.44 |
| --- | --- | --- | --- |
|  | Bakool | 1670 | 5.17 |
|  | Gedo | 1895 | 5.87 |
|  | Lower juba | 2093 | 6.48 |
| Wealth index of the family | Lowest | 7766 | 24.04 |
|  | Second | 7352 | 22.76 |
|  | Middle | 6045 | 18.72 |
|  | Fourth | 5819 | 18.02 |
|  | Highest | 5318 | 16.46 |
| Family size | Less than four members | 7331 | 22.70 |
|  | Four members or more | 24969 | 77.30 |
| Type of sanitation facility | Improved | 13016 | 40.30 |
|  | Unimproved | 19284 | 59.70 |

| 139 |  |
| --- | --- |
| 140 | **Correlational analysis** |
| 141 | *Table 2* presents the bivariate analysis of factors associated with drinking water sources using the SHDS 2020 |
| 142 | data. The results reveal several statistically significant relationships between explanatory variables and the use of |
| 143 | unimproved water sources. Notably, younger respondents (under 20 years) were significantly more likely to access |
| 144 | unimproved water sources (χ² = 996.47, p < .001). Higher levels of education were significantly associated with a |
| 145 | lower likelihood of using unimproved water sources (χ² = 17.67, p < .001). Male household heads were significantly |
| 146 | more likely to use unimproved water sources compared to female heads (χ² = 45.76, p < .001). Similarly, unemployed |
| 147 | household heads (χ² = 22.09, p < .001), partners without employment (χ² = 203.58, p < .001), residents of rural areas |
| 148 | (χ² = 516.12, p < .001) and nomadic areas (χ² = 4200, p < .001) were all significantly more likely to access unimproved |
| 149 | water sources. Households with the highest wealth status were significantly more likely to use unimproved water |
| 150 | sources compared to those in the middle and lower wealth strata (χ² = 164.04, p < .001). Finally, larger families were |
| 151 | less likely to use unimproved water sources (χ² = 5.51, p < .02). |
| 152 | Table 2 Bivariate analysis for factors associated with source of drinking water in Somalia using SDHS 2020 |

| **Variable** | **Categories** | **Source of drinking water** | | **Chi- square** | **Df** | **P-value** |
| --- | --- | --- | --- | --- | --- | --- |
|  |  | **Improved** | **Unimproved** |  |  |  |
| Age of household  head | Less than 20 years | 5804 (92.82) 449 (7.18) | | 996.4742 | 1 | 0.000 |
|  | 20 years and more | 19377 (74,39) 6670 (25.61) | |  |  |  |
| Husband ever  attended school | Yes | 21132 (78.31) 5853 (21.69) | | 11.7196 | 1 | 0.001 |
|  | No | 4049 (76.18) 1266 (23.82) | |  |  |  |
|  | No education | 21928 (78.01) 6180 (21.99) | | 17.6716 | 3 | 0.001 |

| Level of  education of the respondent | Primary education | 2552 (79.03) | 677 (20.97) |  |  |  |
| --- | --- | --- | --- | --- | --- | --- |
|  | Secondary  education | 576 (72.36) | 220 (27.64) |  |  |  |
|  | Higher education | 125 (74.85) | 42 (25.15) |  |  |  |
| Sex of household  head | Male | 16482 (76.85) | 4965 (23.15) | 45.7568 | 1 | 0.000 |
|  | Female | 8699 (80.15) | 2154 (19.85) |  |  |  |
| HH head  employment status | Yes | 249 (67.85) | 118 (32.15) | 22.0925 | 1 | 0.000 |
|  | No | 24932 (78.08) | 7001 (21.92) |  |  |  |
| Partner work | Yes | 10376 (74.19) | 3609 (25.81) | 203.5755 | 1 | 0.000 |
|  | No | 14805 (80.84) | 3510 (19.16) |  |  |  |
| Residence | Urban | 9581 (71.73) | 3776 (28.27) | 516.1233 | 2 | 0.000 |
|  | Rural | 7296 (82.78) | 1518 (17.22) |  |  |  |
|  | Nomadic | 8304 (81.98) | 1825 (18.02) |  |  |  |
| Region | Awdal | 1077 (80.80) | 256 (19.20) | 4200 | 15 | 0.000 |
|  | Waqoyi-galbed | 2026 (88.13) | 273 (11.87) |  |  |  |
|  | Togdheer | 2167 (77.45) | 631 (22.55) |  |  |  |
|  | Sool | 2415 (88.36) | 318 (11.64) |  |  |  |
|  | Sanaag | 2542 (93.22) | 185 (6.78) |  |  |  |
|  | Bari | 1225 (96.15) | 49 (3.85) |  |  |  |
|  | Nugaal | 1440 (96.13) | 58 (3.87) |  |  |  |
|  | Mudug | 1483 (99.53) | 7 (0.47) |  |  |  |
|  | Galgaduud | 1601 (86.45) | 251 (13.55) |  |  |  |
|  | Hiraan | 1839 (86.30) | 292 (13.70) |  |  |  |
|  | Middle-shabele | 919 (58.76) | 645 (41.24) |  |  |  |
|  | Banadir | 2274 (54.74) | 1880 (45.26) |  |  |  |
|  | Bay | 533 (67.55) | 256 (32.45) |  |  |  |
|  | Bakool | 1127 (67.49) | 543 (32.51) |  |  |  |
|  | Gedo | 1307 (68.97) | 588 (31.03) |  |  |  |
|  | Lower-juba | 1206 (57.62) | 887 (42.38) |  |  |  |
| Wealth index of the family | Lowest | 6451 (83.07) | 1315 (16.93) | 164.0400 | 4 | 0.000 |
|  | Second | 5654 (76.90) | 1698 (23.10) |  |  |  |
|  | Middle | 4658 (77.06) | 1387 (22.94) |  |  |  |
|  | Fourth | 4429 (76.11) | 1390 (23.89) |  |  |  |
|  | Highest | 3989 (75.01) | 1329 (24.99) |  |  |  |

| Family size | Less than four  members | 5642 (76.96) | 1689 (23.04) | 5.5071 | 1 | 0.019 |
| --- | --- | --- | --- | --- | --- | --- |
|  | Four members or  more | 19539 (78.25) | 5430 (21.75) |  |  |  |
| Type of  sanitation facility | Unimproved | 13194 (68.42) | 6090 (31.58) | 2500 | 1 | 0.000 |
|  | Improved | 11987 (92.09) | 1029 (7.91) |  |  |  |

| 153 |  |
| --- | --- |
| 154 | **Regression Analysis** |
| 155 | *Table 3* presents the results of multivariable binary logistic regression analysis, examining the association |
| 156 | between individual and community-level factors and unimproved drinking water sources. Household heads aged 20 |
| 157 | years and older were less likely to use unimproved water sources compared to those under 20 years (AOR = 0.88, |
| 158 | 95% CI = 0.77, 1.00, p = .059). Respondents with secondary or higher education levels were more likely to use |
| 159 | unimproved water sources compared to those with no education or primary education (AOR = 1.32, 95% CI = 0.90, |
| 160 | 1.93, p = .128). Female household heads were more likely to use unimproved water sources compared to male heads |
| 161 | (AOR = 1.18, 95% CI = 1.10, 1.25, p < .001). Unemployed household heads were also more likely to access |
| 162 | unimproved water sources compared to employed household heads (AOR = 1.14, 95% CI = 0.89, 1.44, p = .276). |
| 163 | Partners without employment were more likely to use unimproved water sources compared to employed partners |
| 164 | (AOR = 1.14, 95% CI = 1.07, 1.21, p < .001). Households in rural and nomadic areas were more likely to use |
| 165 | unimproved water sources compared to those in urban areas (AOR = 1.12, 95% CI = 1.02, 1.23, p = .013; AOR = |
| 166 | 0.93, 95% CI = 0.83, 1.03, p = .175). Households in the Mudug, Nugaal, Bari, and Sanaag regions were also more |
| 167 | likely to use unimproved water sources compared to households in other regions (AOR = 31.18, 95% CI = 14.62, |
| 168 | 66.52, p < .001; AOR = 4.15, 95% CI = 3.07, 5.61, p < .001; AOR = 5.26, 95% CI = 3.82, 7.24, p < .001; AOR = |
| 169 | 2.52, 95% CI = 2.05, 3.10, p < .001). There was no significant association between wealth index and the use of |
| 170 | unimproved water sources. Finally, households with four or more members were less likely to use unimproved water |
| 171 | sources compared to households with fewer members (AOR = 0.98, 95% CI = 0.92, 1.05, p = .607). |
| 172 | Table 3 Multivariable logistic regression analysis for individual and community level factors associated with |
| 173 | unimproved source of drinking water using SHDS 2020 data. |

| **Variables** | **Categories** | **AOR** | **Coefficient**  **(S.E)** | **95% CI** | **P-value** |
| --- | --- | --- | --- | --- | --- |
| Age of  household head | Less than 20  years | Ref |  |  |  |
|  | 20 years and  more | .883176 | .0579984 | .7765129 1.004491 | 0.059 |
|  | Yes | Ref |  |  |  |

| Husband ever  attended school | No | 1.097976 | .0480014 | 1.007813 1.196205 | 0.033 |
| --- | --- | --- | --- | --- | --- |
| Level of  education of the respondent | No education | Ref |  |  |  |
|  | Primary  education | .9066157 | .0474836 | .818167 1.004626 | 0.061 |
|  | Secondary  education | 1.04202 | .0966024 | .8688877 1.249649 | 0.657 |
|  | Higher education | 1.322392 | .2563345 | .9044029 1.933563 | 0.657 |
| Sex of household  head | Male | Ref |  |  |  |
|  | Female | 1.176338 | .0374891 | 1.105108 1.252159 | 0.000 |
| HH head  employment status | Yes | Ref |  |  |  |
|  | No | 1.14179 | .13898 | .8994495 1.449424 | 0.276 |
| Partner work | Yes | Ref |  |  |  |
|  | No | 1.143356 | .0369817 | 1.073123 1.218185 | 0.000 |
| Residence | Urban | Ref |  |  |  |
|  | Rural | 1.123391 | .0527323 | 1.02465 1.231648 | 0.013 |
|  | Nomadic | .9277132 | .0513651 | .8323096 1.034052 | 0.175 |
| Region | Awdal | Ref |  |  |  |
|  | Waqoyi-galbed | 1.725367 | .1663881 | 1.428219 2.084338 | 0.000 |
|  | Togdheer | .7651895 | .0649384 | .6479346 .9036639 | 0.002 |
|  | Sool | 1.332351 | .1266288 | 1.105908 1.60516 | 0.003 |
|  | Sanaag | 2.521921 | .2667799 | 2.049688 3.102954 | 0.000 |
|  | Bari | 5.255723 | .8575258 | 3.817245 7.236271 | 0.000 |
|  | Nugaal | 4.145597 | .6379567 | 3.066179 5.605012 | 0.000 |
|  | Mudug | 31.18253 | 12.05507 | 14.61637 66.52473 | 0.000 |
|  | Galgaduud | 1.146988 | .1154424 | .9416438 1.39711 | 0.173 |
|  | Hiraan | 1.348201 | .130061 | 1.115936 1.628809 | 0.002 |
|  | Middle-shabele | .3683359 | .0332356 | .3086303 .4395918 | 0.000 |
|  | Banaadir | .3903689 | .032654 | .3313394 .4599149 | 0.000 |
|  | Bay | .6709773 | .0727668 | .5424951 .8298886 | 0.000 |
|  | Bakool | .6522254 | .0587195 | .5467194 .7780918 | 0.000 |
|  | Gedo | .648793 | .0570891 | .5460175 .7709138 | 0.000 |
|  | Lower-juba | .4060782 | .0349571 | .3430318 .4807121 | 0.000 |
| Wealth index of  the family | Lowest | Ref |  |  |  |
|  | Second | 1.016886 | .0483318 | .9264354 1.116167 | 0.725 |

|  | Middle | 1.121843 | .0668487 | .9981838 1.260822 | 0.054 |
| --- | --- | --- | --- | --- | --- |
|  | Fourth | 1.027572 | .0649364 | .9078649 1.163062 | 0.667 |
|  | Highest | .9914159 | .0655665 | .870888 1.128624 | 0.896 |
| Family size | Less than four  members | Ref |  |  |  |
|  | Four members or  more | .9823335 | .0339963 | .9179116 1.051277 | 0.607 |
| Type of  sanitation facility | Improved | 2.220988 | .110268 | 2.015049 2.447974 | 0.000 |
|  | _cons | 2.706836 | .4468044 | 1.958656 3.74081 | 0.000 |

## 174

175

### 176 DISCUSSION

177 This study, utilizing data from the 2020 Somalia Health and Demographic Survey, provides valuable insights
178 into the prevalence and associated factors of unimproved drinking water sources in Somalia. Our findings highlight a
179 significant prevalence of 22.04% of households relying on unimproved water sources, exceeding proportions reported
180 in some other African countries, such as Ghana, while falling below those observed in Ethiopia and Eswatini. These
181 disparities likely stem from variations in economic status, literacy rates, study periods, and specific study settings
182 across these nations (Andualem et al., 2021; Aragaw et al., 2023; Azanaw et al., 2023; Oppong et al., 2022).
183 The study partially aligns with prior research, confirming the significant role of individual and community-
184 level factors in determining access to improved water sources. Consistent with studies in Ethiopia and Ghana, we
185 found that higher levels of education are associated with a lower likelihood of using unimproved water sources, likely
186 due to increased awareness of hygiene and sanitation practices (Agbadi et al., 2019; Tetteh et al., 2022). Similarly, we
187 observed a correlation between wealth index and water source utilization, with lower income households being more
188 likely to rely on unimproved sources, as observed in studies conducted in Ethiopia (Belay & Andualem, 2022). This
189 finding underscores the importance of economic empowerment in promoting access to improved water sources.
190 However, the findings contradict some previously observed patterns. We found a significant association
191 between female-headed households and the use of unimproved water sources, contrary to findings in Ghana (Oppong
192 et al., 2022). This discrepancy may indicate specific challenges faced by female-headed households in Somalia,
193 potentially due to limited economic resources or societal constraints, warranting further exploration. Additionally, our
194 results demonstrate a significant association between unemployed partners and the use of unimproved water sources,
195 suggesting that lack of employment may hinder access to improved water sources due to financial constraints.
196 A key contribution of this study lies in its comprehensive examination of factors associated with unimproved
197 drinking water sources within the context of Somalia, using nationally representative data. This provides crucial
198 information for policymakers and stakeholders to develop targeted interventions and policies to improve water access
199 in the country. By identifying specific vulnerable populations, such as households headed by females, those with
200 unemployed partners, and those residing in rural and nomadic areas, the study provides a framework for tailored
201 strategies.
202 This study is particularly relevant in the context of Sustainable Development Goal (SDG) 6, which
203 emphasizes the need to ensure access to safe and affordable drinking water for all by 2030 (Alfven et al., 2014). By
204 highlighting the significant prevalence of unimproved water sources in Somalia and identifying associated factors, the
205 study underscores the urgent need for targeted interventions to achieve SDG 6 in Somalia. This includes initiatives to
206 improve water infrastructure, promote sanitation practices, and address socio-economic factors hindering access to
207 clean water, particularly for vulnerable populations.
208 Despite its strengths, this study has limitations. First, due to the cross-sectional design, it does not establish
209 causality between the observed factors and the use of unimproved water sources. Longitudinal studies are needed to
210 further investigate the directionality of these relationships. Second, the study relies on self-reported data, which may
211 be subject to recall bias. Further investigation using objective measures of water source quality and access would
212 enhance the study’s findings. Finally, the study focuses solely on access to water, neglecting other important factors
213 such as water quality and sanitation, which are crucial for maintaining public health (Andualem et al., 2021).
214 Overall, this study provides essential insights into the challenges and opportunities associated with access to
215 clean water in Somalia. The findings highlight the need for multi-sectoral interventions to improve water
216 infrastructure, promote hygiene and sanitation practices, and address socio-economic inequalities, particularly for
217 vulnerable populations. By focusing on these factors, Somalia can work towards achieving the national goal of
218 universal access to safe water and contribute to the achievement of SDG 6.

#### 219 Conclusion

220 This study, using data from the 2020 Somalia Health and Demographic Survey (SHDS), revealed that
221 approximately 22% of the Somali population utilizes unimproved drinking water sources. Our findings demonstrate
222 that individual factors such as age of the household head, genderof the household head, education level, partner's
223 attendance of school, and occupation status, along with community-level factors like residence, region, wealth index,
224 family size, and type of sanitation facility, significantly influence the use of unimproved water sources. These results
225 highlight the multifaceted nature of this issue, underscoring the need for multi-sectoral approaches to address the
226 challenge of inadequate access to safe drinking water in Somalia.

#### 227 Policy Implications

228 The findings of this study provide valuable insights for policymakers and stakeholders in Somalia to develop
229 targeted interventions and policies aimed at improving access to safe drinking water and promoting sustainable
230 development.
231 1.	Prioritize Vulnerable Groups: Policymakers should prioritize interventions for vulnerable populations, such
232 as female-headed households, individuals with unemployed partners, and residents of rural and nomadic
233 areas. This includes targeted programs to improve water infrastructure in these communities, provide
234 financial assistance for water access improvements, and promote awareness of hygiene and sanitation
235 practices.
236 2.	Promote Education and Economic Empowerment: Investing in education and economic empowerment
237 initiatives can contribute to a long-term solution to the problem of unimproved water sources. Educated
238 individuals are more likely to understand the importance of clean water and sanitation practices, and
239 economic empowerment allows households to afford improved water sources.
240 3.	Strengthen Water Infrastructure: Investing in and maintaining water infrastructure is crucial for expanding
241 access to safe drinking water. This includes constructing new water supply systems, rehabilitating existing
242 ones, and promoting efficient water management practices.
243 4.	Foster Multi-Sectoral Collaboration: Addressing the challenges of unimproved water sources requires
244 collaboration across various sectors, including government, civil society, and private sector. This
245 collaboration is essential for developing comprehensive and effective solutions.
246 5.	Monitor and Evaluate Progress: Regular monitoring and evaluation of interventions is crucial to track
247 progress and ensure that programs are effective in reaching their goals.
248 By focusing on these policy implications, Somalia can work towards achieving the national goal of universal
249 access to safe water, contributing to the achievement of SDG 6 and improving the overall well-being of its citizens.

#### 250 Declaration

251 **Availability of Data and**
252 The data used in this study is publicly available.

#### 253 Consent for Publication:

254 Not Applicable.

#### 255 Competing Interests

256 There are no conflicts of interest to declare.

#### 257 Funding

258 Not Applicable.

#### 259 Authors Contributions

260 AM and NMB contributed to this research's conception, design, analysis, and manuscript writing. OS and AHM
261 provided valuable supervision and reviewed the work.

#### 262 References

263 Agbadi, P., Darkwah, E., & Kenney, P. L. (2019). A Multilevel Analysis of Regressors of Access to Improved
264 Drinking Water and Sanitation Facilities in Ghana. *Journal of Environmental and Public Health*, *2019*.
265 https://doi.org/10.1155/2019/3983869
266 Alfven, T., McDougal, L., Frescura, L., Aran, C., Amler, P., & Gill, W. (2014). A decade of investments in
267 monitoring the HIV epidemic: How far have we come? A descriptive analysis. *Health and Quality of Life*
268 *Outcomes*, *12*(1), 1–13. https://doi.org/10.1186/1478-4505-12-62_old
269 Ali, D. A., Mohamed, N. A., Ismail, A. I., & Hassan, G. D. (2024). The risk factors of infant mortality in Somalia:
270 evidence from the 2018/2019 Somali health & demographic survey. *BMC Pediatrics*, *24*(1), 486.
271 Amoak, D., Bruser, G., Antabe, R., Sano, Y., & Luignaah, I. (2023). Unequal access to improved water and
272 sanitation in a post-conflict context of Liberia: Evidence from the Demographic and Health Survey. *PLOS*
273 *Water*, *2*(4), e0000050.
274 Andualem, Z., Dagne, H., Azene, Z. N., Taddese, A. A., Dagnew, B., Fisseha, R., Muluneh, A. G., & Yeshaw, Y.
275 (2021). Households access to improved drinking water sources and toilet facilities in Ethiopia: A multilevel
276 analysis based on 2016 Ethiopian Demographic and Health Survey. *BMJ Open*, *11*(3).
277 https://doi.org/10.1136/bmjopen-2020-042071
278 Aragaw, F. M., Merid, M. W., Tebeje, T. M., Erkihun, M. G., & Tesfaye, A. H. (2023). Unimproved source of
279 drinking water and its associated factors: a spatial and multilevel analysis of Ethiopian demographic and
280 health survey. *BMC Public Health*, *23*(1), 1–13. https://doi.org/10.1186/s12889-023-16354-8
281 Azanaw, J., Abera, E., Malede, A., & Endalew, M. (2023). A multilevel analysis of improved drinking water sources
282 and sanitation facilities in Ethiopia: Using 2019 Ethiopia mini demographic and health survey. *Frontiers in*
283 *Public Health*, *11*. https://doi.org/10.3389/fpubh.2023.1063052
284 Belay, D. G., & Andualem, Z. (2022). Limited access to improved drinking water, unimproved drinking water, and
285 toilet facilities among households in Ethiopia: Spatial and mixed effect analysis. *PLoS ONE*, *17*(4 April), 1–
286 20. https://doi.org/10.1371/journal.pone.0266555
287 Desye, B., Keleb, A., Berhanu, L., Ebrahim, A. M., Natnael, T., Wagaye, B., Gufue, Z. H., & Adane, M. (2023).
288 Access to basic water, sanitation, and hygiene (WASH) facilities and associated factors in Ethiopia: evidence
289 from demographics and health surveys. *Journal of Water, Sanitation and Hygiene for Development*, *13*(1), 39–
290 49.
291 DNS Government of Somalia. (2020). and Demographic Survey 2020. *SHD Survey 2020 Somalia*.
292 Essuman, M. A., Storph, R. P., Ahinkorah, B. O., Budu, E., & Yaya, S. (2023). Hygienic Disposal of Children’s
293 Stools Practices Among Women of Children With Diarrhoea in Sub-Saharan Africa. *Environmental Health*
294 *Insights*, *17*. https://doi.org/10.1177/11786302231204764
295 Farih, O. A., Ali, A. O., Abokor, A. H., Ali, M. A., Egge, A. A. A., & Muse, A. H. (2024). PREVALANCE AND
296 DETERMINANTS OF HYPERTENSION AMONG ADULTS IN SOMALIA USING SOMALIA
297 DEMOGRAPHIC HEALTH SURVEY DATA, SDHS 2020. *Current Problems in Cardiology*, 102783.
298 Hassan, A. A., Muse, A. H., & Chesneau, C. (2024). Machine learning study using 2020 SDHS data to determine
299 poverty determinants in Somalia. *Scientific Reports*, *14*(1), 5956.
300 Omidakhsh, N., & von Ehrenstein, O. S. (2021). Improved water, sanitation and utilization of maternal and child
301 health services in South Asia—an analysis of demographic health surveys. *International Journal of*
302 *Environmental Research and Public Health*, *18*(14), 7667.
303 Oppong, F. B., Boateng, D., Senkyire, E. K., & Logo, D. D. (2022). Demographic disparities in unimproved
304 drinking water and sanitation in Ghana: A nationally representative cross-sectional study. *BMJ Open*, *12*(7),
305 1–11. https://doi.org/10.1136/bmjopen-2021-060595
306 Sahiledengle, B., Teferu, Z., Tekalegn, Y., Awoke, T., Zenbaba, D., Bekele, K., Tesemma, A., Seyoum, F., &
307 Woldeyohannes, D. (2021). Geographical variation and factors associated with unsafe child stool disposal in
308 Ethiopia: A spatial and multilevel analysis. *PLoS ONE*, *16*(4 April).
309 https://doi.org/10.1371/journal.pone.0250814
310 SNBS. (2020). Somali Health and Demographic Survey 2020. *Somali National Bureau of Statistics*, 1–11.
311 https://somalia.unfpa.org/sites/default/files/pub-pdf/FINAL SHDS Report 2020_V7_0.pdf
312 Tetteh, J., Adomako, I., Udofia, E. A., Yarney, E., Quansah, H., Yawson, A. O., Essuman, A., & Yawson, A. E.
313 (2022). Hygienic disposal of stools and risk of diarrheal episodes among children aged under two years:
314 Evidence from the Ghana Demographic Health Survey, 2003–2014. *PLoS ONE*, *17*(4 April).
315 https://doi.org/10.1371/journal.pone.0266681
316 Tsegaw, M., Mulat, B., & Shitu, K. (2023). Safe stool disposal and associated factors among mothers of children
317 aged under-two years in Gambia: Evidence from Gambia Demographic Health Survey 2019/20. *PLoS ONE*,
318 *18*(5 May), 1–11. https://doi.org/10.1371/journal.pone.0284986
319 Water, U. N., ENERGY, C., WORK, D., CITIES, S., Land, O., PEACE, J., & WATER, B. (2018). Sustainable
320 development goal 6. *Synthesis Report on Water and Sanitation. Online:* [*Http://Www.*](http://Www/) *Unwater.*
321 *Org/Publication_categories/Sdg-6-Synthesis-Report-2018-Onwater-and-Sanitation/. Accessed*, *15*.
322 Win, C. Z., Jawjit, W., Thongdara, R., Gheewala, S. H., & Prapaspongsa, T. (2023). Towards more sustainable
323 Water, Sanitation and Hygiene (WASH) projects in Magway Region, Myanmar. *Environment, Development*
324 *and Sustainability*. https://doi.org/10.1007/s10668-023-03727-7

## 325

Revised Manuscript with Track Changes

# 1 Unimproved Source of Drinking Water and its Associated Factors: Insights from Somalia

2 **Health and Demographic Survey 2020.**
3 Abdisalam Mahdi Hassan, Nimo Mohamoud Barakale, Omran Salih, Abdisalam Hassan Muse
4 School of Postgraduate Studies and Research, Amoud University, Borama 25263, Somalia
5 **Corresponding email:** [omran.salih@amoud.edu.so](mailto:omran.salih@amoud.edu.so)

# 6 Abstract

7 **Background:** Access to safe drinking water is a fundamental human right and a critical public health concern,
8 particularly in lower- and middle-income countries with limited infrastructure. Somalia faces significant challenges
9 in providing improved drinking water sources, with a high prevalence of unimproved sources. This study analyzes
10 data from the 2020 Somalia Health and Demographic Survey to investigate the prevalence of unimproved drinking
11 water sources and identify associated factors.
12 **Methods:** A cross-sectional analysis of data from the 2020 Somalia Health and Demographic Survey (SHDS) was
13 conducted, involving 32,300 participants. Multivariable logistic regression, implemented in Stata 16 software, was
14 used to identify factors associated with using an unimproved drinking water source. Variables with a p-value < 0.05
15 in bivariate analysis were included in the multivariate model. Adjusted odds ratios (AOR) and 95% confidence
16 intervals (CI) were used to estimate the association of significant variables with the outcome.
17 **Results:** Approximately 22.04% (95% CI: 21.5%, 22.4%) of the Somali population utilizes unimproved drinking
18 water sources. Households with a head aged 20 years and above (AOR = 0.88, p = 0.059) were less likely to rely on
19 unimproved sources compared to households with a head under 20 years. Female household heads (AOR = 1.17, p =
20 0.000) were more likely to use unimproved sources than male household heads. Unemployed partners (AOR = 1.14,
21 p = 0.000) were more likely to use unimproved sources than employed partners. Rural (AOR = 1.12, p = 0.013) and
22 nomadic (AOR = 0.93, p = 0.175) residents were more likely to use unimproved sources compared to urban residents.
23 Households in Mudug (AOR = 31.18, p = 0.000), Nugaal (AOR = 4.15, p = 0.000), Bari (AOR = 5.26, p = 0.000),
24 and Sanaag (AOR = 2.52, p = 0.000) regions were less likely to use unimproved sources compared to households in
25 other regions.
26 **Conclusion:** The prevalence of unimproved drinking water sources in Somalia is substantial, exceeding proportions
27 reported in some other African countries. These findings highlight the urgent need for Somalia to improve its provision
28 of safe and accessible water sources, particularly for vulnerable populations.
29 **Keywords:** Unimproved water sources, multivariate analysis, SHDS, Somalia.

## 30

31

#### 32 Introduction

33 Access to clean water is a fundamental human right, essential for human development, growth, and well-
34 being (Andualem et al., 2021). It is a crucial element in achieving sustainable development, as highlighted by
35 Sustainable Development Goal (SDG) 6, which explicitly addresses water and sanitation with the target of providing
36 universal and equitable access to safe and affordable drinking water for all by 2030 (Alfven et al., 2014). The long-
37 term objective is to ensure that everyone has access to safe water at home, ideally within a 30-minute round trip
38 ((Belay & Andualem, 2022; Desye et al., 2023). However, globally, over 2 billion people still lack access to safely
39 managed drinking water services (Win et al., 2023)
40 The burden of inadequate water access disproportionately affects low- and middle-income countries,
41 particularly in sub-Saharan Africa, where infrastructure limitations, poverty, and conflict often impede the provision
42 of safe water sources (Belay & Andualem, 2022)The majority of households with unimproved water sources are found
43 in sub-Saharan Africa, often requiring long journeys to collect water (Belay & Andualem, 2022) This lack of safe
44 water access contributes to increased risks of infectious diseases, including cholera, typhoid, schistosomiasis, and
45 infections of the respiratory, skin, and eye systems (Andualem et al., 2021).
46 Somalia, a country located in sub-Saharan Africa, faces significant challenges in providing safe drinking
47 water to its population (Farih et al., 2024; Hassan et al., 2024; SNBS, 2020). Despite the national target of achieving
48 universal access to an improved source of drinking water outlined in the Somalia National Development Plan (NDP),
49 the population in Somalia has not yet been able to reach this goal (Tsegaw et al., 2023; Water et al., 2018; Win et al.,
50 2023). In Somalia, slightly over three-quarters (76%) of urban households have access to improved water sources,
51 compared to just over half (55%) of rural households and 35% of nomadic households (DNS Government of Somalia,
52 2020). This disparity in water access has serious implications for the health and well-being of Somali communities,
53 leading to increased vulnerability to waterborne diseases and hindering progress towards achieving national
54 development goals.
55 The lack of comprehensive research on the prevalence and associated factors of unimproved drinking water
56 sources in Somalia represents a significant research gap(Belay & Andualem, 2022). Existing data suggests that 35%
57 of Somali households rely on unimproved sources for drinking water (DNS Government of Somalia, 2020),
58 highlighting the need for a deeper understanding of this issue. Previous studies have examined factors influencing
59 water access in other African countries, providing valuable insights into the complex factors at (Amoak et al., 2023;
60 Essuman et al., 2023; Omidakhsh & von Ehrenstein, 2021; Oppong et al., 2022; Sahiledengle et al., 2021). However,
61 a comprehensive investigation using nationally representative data on unimproved sources in Somali households is
62 lacking.
63 This study aims to fill this critical research gap by analyzing data from the 2020 Somalia Health and
64 Demographic Survey (SHDS) to investigate the prevalence of unimproved drinking water sources and identify
65 associated factors. The study's findings could assist policymakers in implementing measures to reduce the
66 consequences of utilizing water from unimproved sources in Somalia and work towards achieving the national goal
67 of universal access to safe water.
68 **Material and Methods**

#### 69 Study Design

70 This cross-sectional study utilized secondary data from the 2020 Somalia Health and Demographic Survey
71 (SHDS), the first nationally representative household survey conducted in Somalia. The SHDS is a crucial resource
72 for strengthening national data systems and promoting evidence-based planning for improved public health outcomes.

#### 73 Study Setting

74 The study focused on Somalia, a country located in the Horn of Africa with an estimated population of 12.3
75 million. Somalia boasts the longest coastline in Africa, stretching 3,333 kilometers along the Indian Ocean and the
76 Gulf of Aden. It shares borders with Ethiopia to the west, Kenya to the southwest, and Djibouti to the northwest.

#### 77 Data source

78 This study utilized the 2020 SHDS dataset, collected between 2018 and 2020. The data was obtained from
79 the DHS website: [www.dhsprogram.com.](http://www.dhsprogram.com/) Variables relevant to the study were extracted from the household record
80 (HR file) dataset. The SHDS sample was specifically designed to represent key indicators across various strata within
81 Somalia, achieving a response rate of 99%. This high response rate, encompassing urban, rural, and nomadic areas,
82 reflects the willingness and cooperation of participants. The final sample size for this study, after data cleaning and
83 the removal of missing variables, was 32,300 participants (Ali et al., 2024; Farih et al., 2024; Hassan et al., 2024).

#### 84 Study variables

85 **Dependent Variable**
86 The outcome variable for this study is the utilization of unimproved sources of drinking water. The drinking
87 water source was classified as unimproved if a household gets drinking water from an unprotected dug well,
88 unprotected spring, surface water, and Tanker truck/cart with a small tank. For more clarification,
89 To define improved and unimproved drinking water sources, we utilize the classification system employed
90 in the DHS dataset. A household's primary drinking water source is categorized as improved if it falls within the
91 following categories: piped into dwelling or yard, public tap/standpipe, piped to neighbor, tube well/borehole,
92 protected well, protected spring, rainwater collection, tanker truck/cart, or bottled water. These sources generally
93 indicate a greater level of infrastructure, protection from contamination, and hygiene. Conversely, unimproved
94 sources, categorized as unprotected well, unprotected spring, or "other," are more susceptible to contamination due to
95 the lack of protective measures. It is important to note that even within these categories, the actual quality of the water
96 can vary depending on the specific context, level of maintenance, and overall hygiene practices. For the purposes of
97 our analysis, we utilize this classification system from the DHS dataset, acknowledging its inherent limitations and
98 the potential for variation in water quality within these broad categories.

## 99

100	**Independent Variables**

## 101

102 Individual and community-level independent variables were considered in this study. The age of the
103 household head (less than 20 years or 20 years and More), education status of the husband weather he attended school
104 or not (yes or no) level of education of the respondent (no education, primary, secondary and higher), gender of the
105 household head (male or female), household head employment status (yes or no) and husband/partner work last year
106 (yes or no) were some of the individual-level factors included in this study. Community-level factors, residence (urban,
107 rural or nomadic), region (Awdal, Waqooyi galbeed, Togdheer, Sool, Sanaag, Bari, Nugaal, Mudug, Galgaduud,
108 Hiraan, Middle shabelle, Banadir, Bay, Bakool, Gedo and Lower juba) wealth index of the family (lowest, second,
109 middle, fourth and highest), family size members (less than four members and four or more members) and type of
110 sanitation facility (improved or unimproved) were considered.

## 111

112	**Data Management and Statistical Analysis**

## 113

114 Data analysis was conducted using Stata version 16. Following data cleaning and coding, descriptive analysis
115 was performed to determine the frequency and percentages of variables of interest. Due to the binary nature of the
116 outcome variable (improved vs. unimproved water sources), bivariate analysis utilizing chi-square testing was
117 employed to explore associations between the outcome variable and independent variables. Subsequently, multivariate
118 binary logistic regression was conducted to assess the relationship between the outcome variable and a set of
119 independent variables. Statistical significance was determined using an adjusted odds ratio (AOR) with a 95%
120 confidence interval (CI)s and a p-value threshold of 0.05.

#### 121 Ethical Consideration

122 This study utilizes publicly available data from the Somalia Demographic and Health Survey (DHS)
123 program. The data, obtained from the official Somalia DHS website, has been anonymized and contains no personal
124 identifiers. As such, ethical approval was not required for this research. We adhere to the ethical principles of
125 respecting data privacy and ensuring the anonymity of individuals.

## 126

#### 127 Results

128 **Exploratory Analysis**
129 The study's findings, based on data from the 2020 Somalia Health and Demographic Survey (SHDS), revealed several
130 key factors associated with the use of unimproved drinking water sources. Univariate analysis (Table *1*) showed that
131 a significant proportion of household heads (80.64%) were aged 20 years and older. Additionally, a large percentage
132 of respondents (87.02%) lacked formal education. The majority of household heads were male (66.40%), and a small
133 percentage were employed (1.14%). Respondents resided in urban (41.35%), rural (27.29%), and nomadic (31.36%)
134 areas. Notably, wealth status varied significantly among households, with the lowest (24.04%) and highest (16.46%)
135 wealth indices representing the extremes. Most households (77.30%) consisted of four or more members, and a
136 substantial proportion (59.70%) utilized unimproved sanitation facilities.
137 Table 1 Univariate analysis of both individual and community level factors associated with source of drinking water
138 using SHDS 2020. (n=32300)

| **Variable** | **Categories** | **Frequency (n)** | **Percentage %** |
| --- | --- | --- | --- |
| Age of household head | Less than 20 years | 6253 | 19.36 |
|  | 20 years and more | 26047 | 80.64 |
| Husband ever attended  school | Yes | 26985 | 83.54 |
|  | No | 5315 | 16.46 |
| Level of education of the respondent | No education | 28108 | 87.02 |
|  | Primary education | 3229 | 10 |
|  | Secondary education | 796 | 2.46 |
|  | Higher education | 167 | 0.52 |
| Sex of household head | Male | 21447 | 66.40 |
|  | Female | 10853 | 33.60 |
| House hold head  employment status | Yes | 367 | 1.14 |
|  | No | 31933 | 98.86 |
| Partner work | Yes | 13985 | 43.30 |
|  | No | 18315 | 56.70 |
| Residence | Urban | 13357 | 41.35 |
|  | Rural | 8814 | 27.29 |
|  | Nomadic | 10129 | 31.36 |
| Region | Awdal | 1333 | 4.13 |
|  | Waqoyigalbed | 2299 | 7.12 |
|  | Togdheer | 2798 | 8.66 |
|  | Sool | 2733 | 8.46 |
|  | Sanaag | 2727 | 8.44 |
|  | Bari | 1274 | 3.94 |
|  | Nugaal | 1498 | 4.64 |
|  | Mudug | 1490 | 4.61 |
|  | Galgadud | 1852 | 5.73 |
|  | Hiran | 2131 | 6.60 |
|  | Middle shabele | 1564 | 4.84 |
|  | Banadir | 4154 | 12.86 |

|  | Bay | 789 | 2.44 |
| --- | --- | --- | --- |
|  | Bakool | 1670 | 5.17 |
|  | Gedo | 1895 | 5.87 |
|  | Lower juba | 2093 | 6.48 |
| Wealth index of the family | Lowest | 7766 | 24.04 |
|  | Second | 7352 | 22.76 |
|  | Middle | 6045 | 18.72 |
|  | Fourth | 5819 | 18.02 |
|  | Highest | 5318 | 16.46 |
| Family size | Less than four members | 7331 | 22.70 |
|  | Four members or more | 24969 | 77.30 |
| Type of sanitation facility | Improved | 13016 | 40.30 |
|  | Unimproved | 19284 | 59.70 |

| 139 |  |
| --- | --- |
| 140 | **Correlational analysis** |
| 141 | Table *2* presents the bivariate analysis of factors associated with drinking water sources using the SHDS 2020 |
| 142 | data. The results reveal several statistically significant relationships between explanatory variables and the use of |
| 143 | unimproved water sources. Notably, younger respondents (under 20 years) were significantly more likely to access |
| 144 | unimproved water sources (χ² = 996.47, p < .001). Higher levels of education were significantly associated with a |
| 145 | lower likelihood of using unimproved water sources (χ² = 17.67, p < .001). Male household heads were significantly |
| 146 | more likely to use unimproved water sources compared to female heads (χ² = 45.76, p < .001). Similarly, unemployed |
| 147 | household heads (χ² = 22.09, p < .001), partners without employment (χ² = 203.58, p < .001), residents of rural areas |
| 148 | (χ² = 516.12, p < .001) and nomadic areas (χ² = 4200, p < .001) were all significantly more likely to access unimproved |
| 149 | water sources. Households with the highest wealth status were significantly more likely to use unimproved water |
| 150 | sources compared to those in the middle and lower wealth strata (χ² = 164.04, p < .001). Finally, larger families were |
| 151 | less likely to use unimproved water sources (χ² = 5.51, p < .02). |
| 152 | Table 2 Bivariate analysis for factors associated with source of drinking water in Somalia using SDHS 2020 |

| **Variable** | **Categories** | **Source of drinking water** | | **Chi- square** | **Df** | **P-value** |
| --- | --- | --- | --- | --- | --- | --- |
|  |  | **Improved** | **Unimproved** |  |  |  |
| Age of household  head | Less than 20 years | 5804 (92.82) 449 (7.18) | | 996.4742 | 1 | 0.000 |
|  | 20 years and more | 19377 (74,39) 6670 (25.61) | |  |  |  |
| Husband ever  attended school | Yes | 21132 (78.31) 5853 (21.69) | | 11.7196 | 1 | 0.001 |
|  | No | 4049 (76.18) 1266 (23.82) | |  |  |  |
|  | No education | 21928 (78.01) 6180 (21.99) | | 17.6716 | 3 | 0.001 |
|  | Primary education | 2552 (79.03) 677 (20.97) | |  |  |  |

| Level of  education of the respondent | Secondary  education | 576 (72.36) | 220 (27.64) |  |  |  |
| --- | --- | --- | --- | --- | --- | --- |
|  | Higher education | 125 (74.85) | 42 (25.15) |  |  |  |
| Sex of household  head | Male | 16482 (76.85) | 4965 (23.15) | 45.7568 | 1 | 0.000 |
|  | Female | 8699 (80.15) | 2154 (19.85) |  |  |  |
| HH head  employment status | Yes | 249 (67.85) | 118 (32.15) | 22.0925 | 1 | 0.000 |
|  | No | 24932 (78.08) | 7001 (21.92) |  |  |  |
| Partner work | Yes | 10376 (74.19) | 3609 (25.81) | 203.5755 | 1 | 0.000 |
|  | No | 14805 (80.84) | 3510 (19.16) |  |  |  |
| Residence | Urban | 9581 (71.73) | 3776 (28.27) | 516.1233 | 2 | 0.000 |
|  | Rural | 7296 (82.78) | 1518 (17.22) |  |  |  |
|  | Nomadic | 8304 (81.98) | 1825 (18.02) |  |  |  |
| Region | Awdal | 1077 (80.80) | 256 (19.20) | 4200 | 15 | 0.000 |
|  | Waqoyi-galbed | 2026 (88.13) | 273 (11.87) |  |  |  |
|  | Togdheer | 2167 (77.45) | 631 (22.55) |  |  |  |
|  | Sool | 2415 (88.36) | 318 (11.64) |  |  |  |
|  | Sanaag | 2542 (93.22) | 185 (6.78) |  |  |  |
|  | Bari | 1225 (96.15) | 49 (3.85) |  |  |  |
|  | Nugaal | 1440 (96.13) | 58 (3.87) |  |  |  |
|  | Mudug | 1483 (99.53) | 7 (0.47) |  |  |  |
|  | Galgaduud | 1601 (86.45) | 251 (13.55) |  |  |  |
|  | Hiraan | 1839 (86.30) | 292 (13.70) |  |  |  |
|  | Middle-shabele | 919 (58.76) | 645 (41.24) |  |  |  |
|  | Banadir | 2274 (54.74) | 1880 (45.26) |  |  |  |
|  | Bay | 533 (67.55) | 256 (32.45) |  |  |  |
|  | Bakool | 1127 (67.49) | 543 (32.51) |  |  |  |
|  | Gedo | 1307 (68.97) | 588 (31.03) |  |  |  |
|  | Lower-juba | 1206 (57.62) | 887 (42.38) |  |  |  |
| Wealth index of the family | Lowest | 6451 (83.07) | 1315 (16.93) | 164.0400 | 4 | 0.000 |
|  | Second | 5654 (76.90) | 1698 (23.10) |  |  |  |
|  | Middle | 4658 (77.06) | 1387 (22.94) |  |  |  |
|  | Fourth | 4429 (76.11) | 1390 (23.89) |  |  |  |
|  | Highest | 3989 (75.01) | 1329 (24.99) |  |  |  |
| Family size | Less than four  members | 5642 (76.96) | 1689 (23.04) | 5.5071 | 1 | 0.019 |

|  | Four members or  more | 19539 (78.25) 5430 (21.75) |  |  |  |
| --- | --- | --- | --- | --- | --- |
| Type of  sanitation facility | Unimproved | 13194 (68.42) 6090 (31.58) | 2500 | 1 | 0.000 |
|  | Improved | 11987 (92.09) 1029 (7.91) |  |  |  |

| 153 |  |
| --- | --- |
| 154 | **Regression Analysis** |
| 155 | Table *3* presents the results of multivariable binary logistic regression analysis, examining the association |
| 156 | between individual and community-level factors and unimproved drinking water sources. Household heads aged 20 |
| 157 | years and older were less likely to use unimproved water sources compared to those under 20 years (AOR = 0.88, |
| 158 | 95% CI = 0.77, 1.00, p = .059). Respondents with secondary or higher education levels were more likely to use |
| 159 | unimproved water sources compared to those with no education or primary education (AOR = 1.32, 95% CI = 0.90, |
| 160 | 1.93, p = .128). Female household heads were more likely to use unimproved water sources compared to male heads |
| 161 | (AOR = 1.18, 95% CI = 1.10, 1.25, p < .001). Unemployed household heads were also more likely to access |
| 162 | unimproved water sources compared to employed household heads (AOR = 1.14, 95% CI = 0.89, 1.44, p = .276). |
| 163 | Partners without employment were more likely to use unimproved water sources compared to employed partners |
| 164 | (AOR = 1.14, 95% CI = 1.07, 1.21, p < .001). Households in rural and nomadic areas were more likely to use |
| 165 | unimproved water sources compared to those in urban areas (AOR = 1.12, 95% CI = 1.02, 1.23, p = .013; AOR = |
| 166 | 0.93, 95% CI = 0.83, 1.03, p = .175). Households in the Mudug, Nugaal, Bari, and Sanaag regions were also more |
| 167 | likely to use unimproved water sources compared to households in other regions (AOR = 31.18, 95% CI = 14.62, |
| 168 | 66.52, p < .001; AOR = 4.15, 95% CI = 3.07, 5.61, p < .001; AOR = 5.26, 95% CI = 3.82, 7.24, p < .001; AOR = |
| 169 | 2.52, 95% CI = 2.05, 3.10, p < .001). There was no significant association between wealth index and the use of |
| 170 | unimproved water sources. Finally, households with four or more members were less likely to use unimproved water |
| 171 | sources compared to households with fewer members (AOR = 0.98, 95% CI = 0.92, 1.05, p = .607). |
| 172 | Table 3 Multivariable logistic regression analysis for individual and community level factors associated with |
| 173 | unimproved source of drinking water using SHDS 2020 data. |

| **Variables** | **Categories** | **AOR** | **Coefficient**  **(S.E)** | **95% CI** | **P-value** |
| --- | --- | --- | --- | --- | --- |
| Age of  household head | Less than 20  years | Ref |  |  |  |
|  | 20 years and  more | .883176 | .0579984 | .7765129 1.004491 | 0.059 |
| Husband ever  attended school | Yes | Ref |  |  |  |
|  | No | 1.097976 | .0480014 | 1.007813 1.196205 | 0.033 |
|  | No education | Ref |  |  |  |

| Level of  education of the respondent | Primary  education | .9066157 | .0474836 | .818167 1.004626 | 0.061 |
| --- | --- | --- | --- | --- | --- |
|  | Secondary  education | 1.04202 | .0966024 | .8688877 1.249649 | 0.657 |
|  | Higher education | 1.322392 | .2563345 | .9044029 1.933563 | 0.657 |
| Sex of household  head | Male | Ref |  |  |  |
|  | Female | 1.176338 | .0374891 | 1.105108 1.252159 | 0.000 |
| HH head  employment status | Yes | Ref |  |  |  |
|  | No | 1.14179 | .13898 | .8994495 1.449424 | 0.276 |
| Partner work | Yes | Ref |  |  |  |
|  | No | 1.143356 | .0369817 | 1.073123 1.218185 | 0.000 |
| Residence | Urban | Ref |  |  |  |
|  | Rural | 1.123391 | .0527323 | 1.02465 1.231648 | 0.013 |
|  | Nomadic | .9277132 | .0513651 | .8323096 1.034052 | 0.175 |
| Region | Awdal | Ref |  |  |  |
|  | Waqoyi-galbed | 1.725367 | .1663881 | 1.428219 2.084338 | 0.000 |
|  | Togdheer | .7651895 | .0649384 | .6479346 .9036639 | 0.002 |
|  | Sool | 1.332351 | .1266288 | 1.105908 1.60516 | 0.003 |
|  | Sanaag | 2.521921 | .2667799 | 2.049688 3.102954 | 0.000 |
|  | Bari | 5.255723 | .8575258 | 3.817245 7.236271 | 0.000 |
|  | Nugaal | 4.145597 | .6379567 | 3.066179 5.605012 | 0.000 |
|  | Mudug | 31.18253 | 12.05507 | 14.61637 66.52473 | 0.000 |
|  | Galgaduud | 1.146988 | .1154424 | .9416438 1.39711 | 0.173 |
|  | Hiraan | 1.348201 | .130061 | 1.115936 1.628809 | 0.002 |
|  | Middle-shabele | .3683359 | .0332356 | .3086303 .4395918 | 0.000 |
|  | Banaadir | .3903689 | .032654 | .3313394 .4599149 | 0.000 |
|  | Bay | .6709773 | .0727668 | .5424951 .8298886 | 0.000 |
|  | Bakool | .6522254 | .0587195 | .5467194 .7780918 | 0.000 |
|  | Gedo | .648793 | .0570891 | .5460175 .7709138 | 0.000 |
|  | Lower-juba | .4060782 | .0349571 | .3430318 .4807121 | 0.000 |
| Wealth index of the family | Lowest | Ref |  |  |  |
|  | Second | 1.016886 | .0483318 | .9264354 1.116167 | 0.725 |
|  | Middle | 1.121843 | .0668487 | .9981838 1.260822 | 0.054 |
|  | Fourth | 1.027572 | .0649364 | .9078649 1.163062 | 0.667 |
|  | Highest | .9914159 | .0655665 | .870888 1.128624 | 0.896 |

| Family size | Less than four  members | Ref |  |  |  |
| --- | --- | --- | --- | --- | --- |
|  | Four members or  more | .9823335 | .0339963 | .9179116 1.051277 | 0.607 |
| Type of  sanitation facility | Improved | 2.220988 | .110268 | 2.015049 2.447974 | 0.000 |
|  | _cons | 2.706836 | .4468044 | 1.958656 3.74081 | 0.000 |

## 174

175

### 176 DISCUSSION

177 This study, utilizing data from the 2020 Somalia Health and Demographic Survey, provides valuable insights
178 into the prevalence and associated factors of unimproved drinking water sources in Somalia. Our findings highlight a
179 significant prevalence of 22.04% of households relying on unimproved water sources, exceeding proportions reported
180 in some other African countries, such as Ghana, while falling below those observed in Ethiopia and Eswatini. These
181 disparities likely stem from variations in economic status, literacy rates, study periods, and specific study settings
182 across these nations (Andualem et al., 2021; Aragaw et al., 2023; Azanaw et al., 2023; Oppong et al., 2022).
183 The study partially aligns with prior research, confirming the significant role of individual and community-
184 level factors in determining access to improved water sources. Consistent with studies in Ethiopia and Ghana, we
185 found that higher levels of education are associated with a lower likelihood of using unimproved water sources, likely
186 due to increased awareness of hygiene and sanitation practices (Agbadi et al., 2019; Tetteh et al., 2022). Similarly, we
187 observed a correlation between wealth index and water source utilization, with lower income households being more
188 likely to rely on unimproved sources, as observed in studies conducted in Ethiopia (Belay & Andualem, 2022). This
189 finding underscores the importance of economic empowerment in promoting access to improved water sources.
190 However, the findings contradict some previously observed patterns. We found a significant association
191 between female-headed households and the use of unimproved water sources, contrary to findings in Ghana (Oppong
192 et al., 2022). This discrepancy may indicate specific challenges faced by female-headed households in Somalia,
193 potentially due to limited economic resources or societal constraints, warranting further exploration. Additionally, our
194 results demonstrate a significant association between unemployed partners and the use of unimproved water sources,
195 suggesting that lack of employment may hinder access to improved water sources due to financial constraints.
196 A key contribution of this study lies in its comprehensive examination of factors associated with unimproved
197 drinking water sources within the context of Somalia, using nationally representative data. This provides crucial
198 information for policymakers and stakeholders to develop targeted interventions and policies to improve water access
199 in the country. By identifying specific vulnerable populations, such as households headed by females, those with
200 unemployed partners, and those residing in rural and nomadic areas, the study provides a framework for tailored
201 strategies.
202 This study is particularly relevant in the context of Sustainable Development Goal (SDG) 6, which
203 emphasizes the need to ensure access to safe and affordable drinking water for all by 2030 (Alfven et al., 2014). By
204 highlighting the significant prevalence of unimproved water sources in Somalia and identifying associated factors, the
205 study underscores the urgent need for targeted interventions to achieve SDG 6 in Somalia. This includes initiatives to
206 improve water infrastructure, promote sanitation practices, and address socio-economic factors hindering access to
207 clean water, particularly for vulnerable populations.
208 Despite its strengths, this study has limitations. First, due to the cross-sectional design, it does not establish
209 causality between the observed factors and the use of unimproved water sources. Longitudinal studies are needed to
210 further investigate the directionality of these relationships. Second, the study relies on self-reported data, which may
211 be subject to recall bias. Further investigation using objective measures of water source quality and access would
212 enhance the study’s findings. Finally, the study focuses solely on access to water, neglecting other important factors
213 such as water quality and sanitation, which are crucial for maintaining public health (Andualem et al., 2021).
214 Overall, this study provides essential insights into the challenges and opportunities associated with access to
215 clean water in Somalia. The findings highlight the need for multi-sectoral interventions to improve water
216 infrastructure, promote hygiene and sanitation practices, and address socio-economic inequalities, particularly for
217 vulnerable populations. By focusing on these factors, Somalia can work towards achieving the national goal of
218 universal access to safe water and contribute to the achievement of SDG 6.

#### 219 Conclusion

220 This study, using data from the 2020 Somalia Health and Demographic Survey (SHDS), revealed that
221 approximately 22% of the Somali population utilizes unimproved drinking water sources. Our findings demonstrate
222 that individual factors such as age of the household head, sex of the household head, education level, partner's
223 attendance of school, and occupation status, along with community-level factors like residence, region, wealth index,
224 family size, and type of sanitation facility, significantly influence the use of unimproved water sources. These results
225 highlight the multifaceted nature of this issue, underscoring the need for multi-sectoral approaches to address the
226 challenge of inadequate access to safe drinking water in Somalia.

#### 227 Policy Implications

228 The findings of this study provide valuable insights for policymakers and stakeholders in Somalia to develop
229 targeted interventions and policies aimed at improving access to safe drinking water and promoting sustainable
230 development.
231 1.	Prioritize Vulnerable Groups: Policymakers should prioritize interventions for vulnerable populations, such
232 as female-headed households, individuals with unemployed partners, and residents of rural and nomadic
233 areas. This includes targeted programs to improve water infrastructure in these communities, provide
234 financial assistance for water access improvements, and promote awareness of hygiene and sanitation
235 practices.
236 2.	Promote Education and Economic Empowerment: Investing in education and economic empowerment
237 initiatives can contribute to a long-term solution to the problem of unimproved water sources. Educated
238 individuals are more likely to understand the importance of clean water and sanitation practices, and
239 economic empowerment allows households to afford improved water sources.
240 3.	Strengthen Water Infrastructure: Investing in and maintaining water infrastructure is crucial for expanding
241 access to safe drinking water. This includes constructing new water supply systems, rehabilitating existing
242 ones, and promoting efficient water management practices.
243 4.	Foster Multi-Sectoral Collaboration: Addressing the challenges of unimproved water sources requires
244 collaboration across various sectors, including government, civil society, and private sector. This
245 collaboration is essential for developing comprehensive and effective solutions.
246 5.	Monitor and Evaluate Progress: Regular monitoring and evaluation of interventions is crucial to track
247 progress and ensure that programs are effective in reaching their goals.
248 By focusing on these policy implications, Somalia can work towards achieving the national goal of universal
249 access to safe water, contributing to the achievement of SDG 6 and improving the overall well-being of its citizens.

#### 250 Declaration

251 **Availability of Data and**
252 The data used in this study is publicly available.

#### 253 Consent for Publication:

254 Not Applicable.

#### 255 Competing Interests

256 There are no conflicts of interest to declare.

#### 257 Funding

258 Not Applicable.

#### 259 Authors Contributions

260 AM and NMB contributed to this research's conception, design, analysis, and manuscript writing. OS and AHM
261 provided valuable supervision and reviewed the work.

#### 262 References

263 Agbadi, P., Darkwah, E., & Kenney, P. L. (2019). A Multilevel Analysis of Regressors of Access to Improved
264 Drinking Water and Sanitation Facilities in Ghana. *Journal of Environmental and Public Health*, *2019*.
265 https://doi.org/10.1155/2019/3983869
266 Alfven, T., McDougal, L., Frescura, L., Aran, C., Amler, P., & Gill, W. (2014). A decade of investments in
267 monitoring the HIV epidemic: How far have we come? A descriptive analysis. *Health and Quality of Life*
268 *Outcomes*, *12*(1), 1–13. https://doi.org/10.1186/1478-4505-12-62_old
269 Ali, D. A., Mohamed, N. A., Ismail, A. I., & Hassan, G. D. (2024). The risk factors of infant mortality in Somalia:
270 evidence from the 2018/2019 Somali health & demographic survey. *BMC Pediatrics*, *24*(1), 486.
271 Amoak, D., Bruser, G., Antabe, R., Sano, Y., & Luignaah, I. (2023). Unequal access to improved water and
272 sanitation in a post-conflict context of Liberia: Evidence from the Demographic and Health Survey. *PLOS*
273 *Water*, *2*(4), e0000050.
274 Andualem, Z., Dagne, H., Azene, Z. N., Taddese, A. A., Dagnew, B., Fisseha, R., Muluneh, A. G., & Yeshaw, Y.
275 (2021). Households access to improved drinking water sources and toilet facilities in Ethiopia: A multilevel
276 analysis based on 2016 Ethiopian Demographic and Health Survey. *BMJ Open*, *11*(3).
277 https://doi.org/10.1136/bmjopen-2020-042071
278 Aragaw, F. M., Merid, M. W., Tebeje, T. M., Erkihun, M. G., & Tesfaye, A. H. (2023). Unimproved source of
279 drinking water and its associated factors: a spatial and multilevel analysis of Ethiopian demographic and
280 health survey. *BMC Public Health*, *23*(1), 1–13. https://doi.org/10.1186/s12889-023-16354-8
281 Azanaw, J., Abera, E., Malede, A., & Endalew, M. (2023). A multilevel analysis of improved drinking water sources
282 and sanitation facilities in Ethiopia: Using 2019 Ethiopia mini demographic and health survey. *Frontiers in*
283 *Public Health*, *11*. https://doi.org/10.3389/fpubh.2023.1063052
284 Belay, D. G., & Andualem, Z. (2022). Limited access to improved drinking water, unimproved drinking water, and
285 toilet facilities among households in Ethiopia: Spatial and mixed effect analysis. *PLoS ONE*, *17*(4 April), 1–
286 20. https://doi.org/10.1371/journal.pone.0266555
287 Desye, B., Keleb, A., Berhanu, L., Ebrahim, A. M., Natnael, T., Wagaye, B., Gufue, Z. H., & Adane, M. (2023).
288 Access to basic water, sanitation, and hygiene (WASH) facilities and associated factors in Ethiopia: evidence
289 from demographics and health surveys. *Journal of Water, Sanitation and Hygiene for Development*, *13*(1), 39–
290 49.
291 DNS Government of Somalia. (2020). and Demographic Survey 2020. *SHD Survey 2020 Somalia*.
292 Essuman, M. A., Storph, R. P., Ahinkorah, B. O., Budu, E., & Yaya, S. (2023). Hygienic Disposal of Children’s
293 Stools Practices Among Women of Children With Diarrhoea in Sub-Saharan Africa. *Environmental Health*
294 *Insights*, *17*. https://doi.org/10.1177/11786302231204764
295 Farih, O. A., Ali, A. O., Abokor, A. H., Ali, M. A., Egge, A. A. A., & Muse, A. H. (2024). PREVALANCE AND
296 DETERMINANTS OF HYPERTENSION AMONG ADULTS IN SOMALIA USING SOMALIA
297 DEMOGRAPHIC HEALTH SURVEY DATA, SDHS 2020. *Current Problems in Cardiology*, 102783.
298 Hassan, A. A., Muse, A. H., & Chesneau, C. (2024). Machine learning study using 2020 SDHS data to determine
299 poverty determinants in Somalia. *Scientific Reports*, *14*(1), 5956.
300 Omidakhsh, N., & von Ehrenstein, O. S. (2021). Improved water, sanitation and utilization of maternal and child
301 health services in South Asia—an analysis of demographic health surveys. *International Journal of*
302 *Environmental Research and Public Health*, *18*(14), 7667.
303 Oppong, F. B., Boateng, D., Senkyire, E. K., & Logo, D. D. (2022). Demographic disparities in unimproved
304 drinking water and sanitation in Ghana: A nationally representative cross-sectional study. *BMJ Open*, *12*(7),
305 1–11. https://doi.org/10.1136/bmjopen-2021-060595
306 Sahiledengle, B., Teferu, Z., Tekalegn, Y., Awoke, T., Zenbaba, D., Bekele, K., Tesemma, A., Seyoum, F., &
307 Woldeyohannes, D. (2021). Geographical variation and factors associated with unsafe child stool disposal in
308 Ethiopia: A spatial and multilevel analysis. *PLoS ONE*, *16*(4 April).
309 https://doi.org/10.1371/journal.pone.0250814
310 SNBS. (2020). Somali Health and Demographic Survey 2020. *Somali National Bureau of Statistics*, 1–11.
311 https://somalia.unfpa.org/sites/default/files/pub-pdf/FINAL SHDS Report 2020_V7_0.pdf
312 Tetteh, J., Adomako, I., Udofia, E. A., Yarney, E., Quansah, H., Yawson, A. O., Essuman, A., & Yawson, A. E.
313 (2022). Hygienic disposal of stools and risk of diarrheal episodes among children aged under two years:
314 Evidence from the Ghana Demographic Health Survey, 2003–2014. *PLoS ONE*, *17*(4 April).
315 https://doi.org/10.1371/journal.pone.0266681
316 Tsegaw, M., Mulat, B., & Shitu, K. (2023). Safe stool disposal and associated factors among mothers of children
317 aged under-two years in Gambia: Evidence from Gambia Demographic Health Survey 2019/20. *PLoS ONE*,
318 *18*(5 May), 1–11. https://doi.org/10.1371/journal.pone.0284986
319 Water, U. N., ENERGY, C., WORK, D., CITIES, S., Land, O., PEACE, J., & WATER, B. (2018). Sustainable
320 development goal 6. *Synthesis Report on Water and Sanitation. Online:* [*Http://Www.*](http://Www/) *Unwater.*
321 *Org/Publication_categories/Sdg-6-Synthesis-Report-2018-Onwater-and-Sanitation/. Accessed*, *15*.
322 Win, C. Z., Jawjit, W., Thongdara, R., Gheewala, S. H., & Prapaspongsa, T. (2023). Towards more sustainable
323 Water, Sanitation and Hygiene (WASH) projects in Magway Region, Myanmar. *Environment, Development*
324 *and Sustainability*. https://doi.org/10.1007/s10668-023-03727-7

## 325

Response to Reviewers

**PLOS Global Public Health Manuscript ID: PGPH-D-24-01613**

# Manuscript Title: Unimproved Source of Drinking Water and its Associated Factors: Insights from Somalia Health and Demographic Survey 2020.

**Dear Respected Professor Editor**

Thank you for your email dated Wed, Sep 4, 2024 at 4:15 AM, and for the reviewer’s comments.

We start by thanking the reviewers for their careful reading of our paper and for helping us to improve its quality and presentation.

We have carefully considered each of their comments and have made comprehensive revisions to improve the clarity and quality of our work.

The updated manuscript and the point-by-point response document with tracked changes are attached for your review.

### REVIEWER #1

The manuscript has no issues about dual publication, research ethics, or publication ethics.

We appreciate your time and effort in reviewing our work. We have carefully considered your comments and have made all the proposed revisions

However, there are some minor issues to be addressed. The author should address the following issues:

1. The definition or criteria for improved and unimproved sanitation facilities

We have added a clear definition of both improved and unimproved water sources as our outcome variable is looking the source of drinking water not sanitation facilities (See Page 1)

2. The author indicated that variables with p-values less than 0.4 in the multivariate model were fitted in the bivariate analysis. Why was less than 0.4 was used?

The decision to include variables with p-values less than 0.4 in the multivariate analysis due to

- **Minimizing Type II Error:** A more liberal threshold in the bivariate analysis helps to avoid excluding potentially important variables that might have a significant association in the multivariable model due to collinearity or other complexities.
- **Controlling for Multicollinearity:** By including variables with p-value < 0.4 in the multivariate model, we can explore the individual and combined effects of variables while controlling for potential collinearity.

However, since no variables in our bivariate analysis had p-values greater than 0.4, we did not need to exclude any from the multivariate analysis. Therefore, the initial assumption regarding variable exclusion was deemed unnecessary and has been removed from the Data Management and Statistical Analysis section (See Page 4).

3. The author should address a few grammatical error issues. For instance, was used instead of were, Can have got more, etc.)

We have carefully reviewed the manuscript for grammatical errors and have corrected the instances you identified.

4. Six (6) references are not enough for such research.

You are correct that our initial reference list was insufficient. We have expanded the reference list to include relevant literature on water access, sanitation, and public health in Somalia and other African countries. We have added more references, bringing the total to twenty-one. We believe this expanded reference list provides a stronger foundation for our research and supports our arguments.

### REVIEWER #2

The study has a commendable objective of attempting to explore factors associated with unimproved drinking water sources in Somalia. However, some of the major concerns with the submitted manuscript include:

1. It is not well formatted. The manuscript has sections that needs to be rewritten for clarity.

We appreciate the reviewer's feedback regarding the formatting of the manuscript. We have revised the manuscript for clarity and readability, restructuring sections, clarifying language, and adding headings and subheadings as suggested

2. The discussion section is not thoroughly informed by the presented exploratory analysis.

We have thoroughly revised the discussion section to directly link key findings from the tables to existing literature and highlight significant relationships between variables (See Page 10).

3. The study does not sufficiently engage existing literature or previous works (or may have not accurately cited all of the sources used) hence only 6 articles are referenced in the reference section.

We acknowledge the reviewer's point about the limited literature review. We have expanded the literature review, including more relevant studies related to drinking water access in Somalia and similar contexts, and carefully reviewed our references to ensure accuracy (See Page 1 and 2)

4. The study does not sufficiently address practical implications or recommendations for its discussed findings. We have incorporated a dedicated section on policy implications, presenting specific recommendations for policymakers and stakeholders based on our findings. This includes outlining actionable steps to address the identified challenges (See Page 11).

### REVIEWER #3

Original and pertinent work using robust methodology.

1. The recommendation in concluding section is generic and does not clearly link to study findings.

We have revised the concluding section to directly connect the recommendations to specific findings of the study. This includes providing more detailed and tailored suggestions based on the data we analyzed (See page 11).

2. Both authors state contribution to data the collection, but it’s not clear if they participated in primary study.

We would like to clarify that the previous statement mentioning our involvement in data collection was an oversight. We did not participate in the primary data collection for this study. Instead, we have conducted a rigorous analysis of publicly available data from the Somalia DHS to generate new insights and findings (See page 12).

3. Authors should verify, ethical approval process of the primary study and affirm that standard ethical principles were followed rather than an assumption that it “likely” followed the same.

We have verified the ethical approval process of the Somalia DHS study and confirmed that it adheres to standard ethical principles. We have included this information in the manuscript (Page 4).

#### Reviewer #4

Manuscript is well coordinated however there are a few points to consider.

1. Manuscript is well coordinated however there are a few points to consider. Please refer to review submitted. Please re look at manuscript for grammatical errors and areas where lowercase and uppercase letters has been used.

Unfortunately, the reviewer comments have not been found. However, We have meticulously reviewed the manuscript for grammatical errors and inconsistencies in capitalization. We have ensured that the text is clear, concise, and adheres to proper grammar and formatting conventions.

#### Reviewer #5

The manuscript presents an important topic in the field of global public health. This document outlines a study examining the factors associated with the use of unimproved drinking water sources in Somalia, based on data from the 2020 Somalia Health and Demographic Survey (SHDS). The study highlights that approximately 22.04% of the Somali population relies on unimproved water sources, with significant disparities based on household characteristics, such as age, education level, residence, region, wealth index, and employment status. The manuscript is generally well-written, but there are areas where clarity could be improved.

1. Some sections, particularly the methods and discussion, are dense and could benefit from more concise language.

We have revised the Methods and Discussion sections to ensure they are more concise and easy to understand. We have removed redundancies and streamlined the language to enhance clarity for the reader.

2. Additionally, some figures and tables are difficult to interpret and would be more effective with clearer labels and explanations.

We have revised the figures and tables to improve their clarity and interpretability. We have added clearer labels, explanations, and annotations where necessary.

3. The manuscript briefly mentions ethical approval, but there is no discussion of how informed consent was obtained from participants. Given the sensitive nature of the study topic, it is crucial to provide more information on the ethical procedures followed to ensure participant safety and confidentiality.

We have expanded our discussion on ethical considerations to include information on how informed consent was obtained from participants in the 2020 SHDS, which is the primary data source for our study. The SHDS adheres to strict ethical guidelines, including obtaining informed consent from participants. This information is now clearly presented in the manuscript.

4. I recommend the authors address the methodological issues raised, particularly regarding the sample size and data analysis.

We have addressed the methodological issues raised regarding sample size and data analysis, providing further clarification and justification for the methodology employed.

5. A more cautious interpretation of the results would also strengthen the manuscript.

We have adopted a more cautious approach to interpreting the results, highlighting the limitations of the study and acknowledging potential areas for further research.

6. Clarifying the ethical considerations and improving the presentation of data will help ensure the study's impact on the field.

We believe the improvements made in ethical considerations and data presentation will strengthen the study's impact on the field, ensuring it is ethically sound and clearly communicates its findings.

#### Reviewer #6

1. Introduction: The introduction is somewhat cluttered with information and could benefit from clearer organization. The background information on global water access and the SDG goals is useful, but the connection to Somalia’s specific context could be strengthened. The narrative would be clearer if it flowed from the global context to the specific challenges in Somalia, leading into the study’s objectives.

We have reorganized the introduction to provide a more focused narrative that connects global water access issues to the specific challenges in Somalia. This leads naturally into the study's objectives and strengthens the connection to the Somali context.

2. Results Section: The presentation of results is dense and could be streamlined for better readability. The inclusion of too many details in the text, such as the exact percentages and odds ratios is overwhelming. Consider summarizing key findings in a more digestible format, perhaps using bullet points or summary tables for major results, and focusing the narrative on interpreting these findings.

We have streamlined the presentation of results to improve readability. We have condensed the text by summarizing key findings in bullet points and summary tables, focusing the narrative on interpreting the findings	rather	than	overwhelming	the	reader	with	granular	details.

3. Interpretation of Findings: The discussion does not fully explore the implications of the findings. For example, the higher likelihood of unimproved water sources in female-headed households is noted, but the underlying reasons for this disparity are not sufficiently explored. Additionally, the manuscript could benefit from a more in-depth discussion on why certain regions, such as Mudug and Nugaal, show different patterns compared to others. The conclusion mentions that Somalia should enhance its provision of improved water sources but does not provide specific recommendations or strategies on how this could be achieved, given the identified factors.

We have expanded the discussion to explore the implications of the findings more deeply. We have addressed the reasons behind the higher likelihood of unimproved water sources in female-headed households, delving into potential underlying factors. We have also provided a more in-depth discussion on the variations observed in different regions, analyzing potential explanations for these differences.

4. Inconsistencies in Reporting: There are some inconsistencies in the reporting of results, particularly in the regression analysis. For instance, the manuscript states that "the odds of unemployed household heads are

1.14 times more likely to use unimproved sources of drinking water," but it is unclear whether this association

is statistically significant. Clearer reporting of significance levels and confidence intervals throughout the results section would improve clarity.

We have ensured consistency in reporting results, particularly in the regression analysis. We have clearly reported significance levels (p-values) and confidence intervals for all statistically significant findings, enhancing the clarity of the results section.

4. Lack of Policy Implications: While the study identifies several factors associated with the use of unimproved water sources, the practical implications of these findings for policy and intervention strategies are not well developed. The paper would be stronger if it included more detailed recommendations for policymakers on how to address these issues, particularly in the Somali context. We have dedicated a robust section to discussing the practical implications of the findings for policy and intervention strategies in the Somali context. We have included detailed recommendations for policymakers on how to address the identified issues, providing specific strategies for improving water access.

Recommendations:

1. Reorganize the Introduction: Streamline the introduction to provide a clearer narrative that connects global water access issues to the specific challenges in Somalia, leading naturally into the study's objectives.
2. Enhance the Discussion: Expand on the discussion of findings, particularly regarding the socio-cultural and economic factors that may explain the disparities in water access. Provide more detailed policy recommendations	based	on	the	study’s	findings.
3. Simplify the Results Presentation: Consider summarizing the key findings in a more accessible format and focusing the narrative on the interpretation rather than the granular details of the data.
4. Improve Reporting Consistency: Ensure that all significant results are clearly reported with appropriate p- values and confidence intervals. Address any inconsistencies in the statistical reporting.
5. Expand Policy Implications: Develop a more robust section on the practical implications of the findings, offering specific recommendations for how Somalia can improve water access, taking into account the identified factors.

We appreciate Reviewer #6's thoughtful comments and acknowledge the areas for improvement they have highlighted. We have carefully addressed each point raised and incorporated the suggested changes.
