# Supplementary material for: Unimproved source of drinking water and the associated factors: Insights from the 2020 Somalia demographic and health survey
Source: PLOS Glob Public Health. 2024 Dec 5;4(12):e0003844. doi: 10.1371/journal.pgph.0003844 (PMC11620610; doi:10.1371/journal.pgph.0003844)
Supplement: Attachment — Submitted filename: Respond to reveiwer.docx [file pgph.0003844.s003.docx]

**Manuscript ID:** PGPH-D-24-01613_R1

**Manuscript Title: Unimproved Source of Drinking Water and the Associated Factors: Insights from Somalia Health and Demographic Survey 2020.**

**To:** **PLOS Global Public Health** Editor

**Re:** Response to reviewers

Dear Editor,

Thank you for allowing a resubmission of our manuscript, with an opportunity to address the reviewers’ comments.

We are uploading (a) our point-by-point response to the comments (below) (response to reviewers), (b) an updated manuscript with yellow highlighting indicating changes, and (c) a clean updated manuscript without highlights.

We look forward to your decision.

Finally, we would like to thank the reviewers for reviewing the paper and providing suggestions to improve the paper’s quality. The authors have addressed the comments and suggestions, as described in the response below.

Best regards,

Authors.

***Journal Requirements***

Please review your reference list to ensure that it is complete and correct. If you have cited papers that have been retracted, please include the rationale for doing so in the manuscript text,or remove these references and replace them with relevant current references. Any changes to the reference list should be mentioned in the rebuttal letter that accompanies your revised manuscript. If you need to cite a retracted article, indicate the article’s retracted status in the References list and also include a citation and full reference for the retraction notice.

**Authors response:** We have made the required review to ensure that the reference list is complete and correct.

**Additional Editor Comments**

**Concern #1:** Please, review the attachment by the Reviewer thoroughly and revise the manuscript accordingly. Very importantly, please revise the manuscript for language correctness and critically improve the English structure.

**Authors response:** We have revised the attachment thoroughly and revised the manuscript accordingly, addressing all comments and suggestions provided by the reviewer **(See all the tables, Study variables section, and conclusion section**).

**Concern #2:** Revise the title as follows: Unimproved Source of Drinking Water and the Associated Factors: Insights from the 2020 Somalia Demographic and Health Survey, instead of the way you initially provided it.

**Authors response:** The suggestions have been addressed as requested **(See the titles**).

**Reviewer #4**

**Concern #1:** Dear authors
Thank you for addressing previous comments. I am attaching a word document with a few track changes, of some items that were missed.
All the best!

**Authors response:** We have revised the attachment thoroughly and revised the manuscript, accordingly, addressing all comments and suggestions provided by the reviewer **(See all the tables, Study variables section, and conclusion section**).

**Thank you**
